# In Silico Pre-Screening as a Design-Assessment Tool for Gold Nanoparticle-Amplified DNA Displacement Assays: Identifying Sequence-Level Design Limitations Across Electrode and Magnetic-Bead Configurations

**DOI:** 10.3390/ijms27198652

**Published:** 2026-09-27

**Authors:** Darius Riziki Martin, Tatchanun Ngamdee, Ruben Cloete, Abram Madimabe Madiehe, Mervin Meyer, Patsamon Rijiravanich

**Affiliations:** 1 Department of Science, Technology and Innovation (DSTI)/TIA Innovation Platform, Department of Biotechnology, University of the Western Cape, Bellville 7535, South Africa; amadiehe@uwc.ac.za; 2 South African National Bioinformatics Institute (SANBI), University of the Western Cape, Bellville 7535, South Africa; ruben@sanbi.ac.za; 3 Sensor Technology Laboratory, Pilot Plant Development and Training Institute, King Mongkut’s University of Technology Thonburi, Bang Khun Thian, Bangkok 10150, Thailand; tatchanun.ngam@gmail.com (T.N.); patsamon.rij@biotec.or.th (P.R.); 4 Nanobiotechnology Research Group, Department of Biotechnology, University of the Western Cape, Bellville 7535, South Africa; 5 Industrial Sensor Technology Research Team, National Center for Genetic Engineering and Biotechnology (BIOTEC), National Science and Technology Development Agency (NSTDA), King Mongkut’s University of Technology Thonburi, Bang Khun Thian, Bangkok 10150, Thailand

**Keywords:** electrochemical DNA biosensor, gold nanoparticles, target-induced strand displacement, DNA immobilisation, molecular docking

## Abstract

The growing demand for decentralised and point-of-care (POC) nucleic acid testing has increased interest in label-free, sequence-specific DNA biosensors and computational approaches that can guide their design before extensive experimental optimisation. Here, an in silico workflow was first employed to evaluate a gold nanoparticle (AuNP)-amplified, target-induced DNA displacement assay. Nearest-neighbour duplex thermodynamics, DNA secondary-structure analysis, and High Ambiguity Driven biomolecular DOCKing (HADDOCK) were used to assess sequence complementarity and displacement behaviour. The analyses confirmed Watson–Crick complementarity among the electrode capture, reporter, and target sequences and identified an unintended mismatch in the magnetic bead-capture probe. HADDOCK analyses using default RNA and explicit DNA settings consistently favoured target over non-target displacement, although control poses were more setting-sensitive. Experimental testing subsequently provided supporting evidence for the computational assessment. Electrochemical measurements showed the anticipated signal hierarchy of target > non-target > buffer, although differences were not statistically significant at the replicate numbers tested. The magnetic bead platform produced weak, non-differential responses, consistent with the sequence-level design limitation identified computationally. Together, these findings demonstrate the utility of computational pre-screening for interrogating displacement mechanisms, identifying correctable design errors, and guiding rational experimental optimisation of nucleic acid biosensors. No statistically significant target discrimination was achieved at the replicate numbers tested, and the proposed corrected bead-capture sequence has not been experimentally validated.

## 1. Introduction

Electrochemical DNA biosensors are a low-cost, rapid, and field-deployable alternative to optical and PCR-based methods for detecting nucleic acids. They offer high sensitivity, simple operation, and good portability [1]. Gold nanoparticles (AuNPs) are widely used as electrochemical labels in these assays. This is because they are easy to make with consistent quality, readily attach to biomolecules, and remain stable in solution. AuNPs are often paired with magnetic bead-based separation to simplify target isolation and purification [1]. Demand for decentralised, point-of-care (POC) nucleic acid testing has continued to grow, and electrochemical platforms remain among the most promising formats for translating laboratory-grade sensitivity into rapid, low-cost, field-deployable devices [2].

Strand displacement assays work as follows. A target sequence competes with and displaces a reporter strand that is already attached to a nanoparticle and is hybridised to an immobilised capture probe. This produces a measurable change in the electrochemical signal that is directly linked to the target binding. It does not rely solely on the direct hybridisation of the target [1]. Displacement can occur in two ways. It can proceed through toehold-mediated branch migration, where the target carries an additional complementary sequence beyond that of the reporter. Alternatively, it can proceed through a concentration-driven mass action exchange, where the target and reporter share the same recognition sequence and displacement depends on their relative concentrations [3]. For example, toehold-mediated strand displacement has recently been combined with AuNP-decorated MXene electrodes to detect the KRAS oncogene down to 0.38 fM without enzymatic amplification, demonstrating the applicability of this approach to cancer biomarker detection [4].

This study builds on a target-induced strand displacement and AuNP labelling framework that members of this collaboration previously developed for viral nucleic acid detection [3]. Here, we extend this framework to a DNA-based model target system, introduce two parallel capture probe immobilisation strategies, and add a structural bioinformatics component that supports and, where needed, corrects the displacement assay design before and alongside experimental testing. This computational component was not included in the prior work of the group. Although electrochemical DNA displacement biosensors have been reported for several nucleic acid biomarkers, including cancer-associated targets [5], few studies have directly compared multiple capture-probe immobilisation chemistries alongside an explicit sequence- and structure-level computational check within the same displacement assay. We therefore compared two immobilisation strategies directly, applying the same sequence-level computational check to both platforms before either wet-lab result was known, so that any design error would be caught by the same standard on both sides of the comparison.

In this study, we compared two immobilisation strategies for the capture probe, DNA1, on screen-printed carbon electrodes. The first strategy used an applied electric current to exploit the negatively charged phosphate backbone of DNA1 under an induced positive surface charge. The second strategy involved passive overnight incubation under humid conditions. Both strategies were tested for their ability to support AuNP-amplified target-induced displacement assays. In this assay, the target sequence, called DNA3, was expected to displace the AuNP-conjugated reporter probe, called DNA2, producing an increase in the measured current relative to the non-target and buffer-only controls.

In addition to the wet lab assay, we used a structural bioinformatics workflow to computationally assess the predicted hybridisation stability of the capture probe and reporter probe duplexes, DNA1 and DNA2, relative to the capture probe and target duplexes, DNA1 and DNA3. This provided a sequence-level basis for the expected displacement behaviour before experimental testing. A similar in silico approach was recently used to truncate and validate an aptamer for an electrochemical okadaic acid sensor before any electrode work began [6]. We took the same approach here, where the capture probe DNA1 was tested under two immobilisation strategies, electric current-assisted deposition and passive overnight incubation, on screen-printed carbon electrodes. These were tested alongside a parallel streptavidin magnetic bead platform, and nearest-neighbour thermodynamics, a DNA secondary structure check, and molecular docking were applied to each platform’s sequences before their wet-lab results were interpreted, so that design errors could be caught early rather than diagnosed after.

The computational analyses reported here served two distinct roles. The sequence-level screening of the electrode capture and reporter sequences (DNA1, DNA2, DNA3) was performed prospectively (before the electrode experiments were conducted), confirming the design was thermodynamically sound before resources were committed. The Bio DNA mismatch was identified during the experimental design phase, providing a diagnostic explanation once the bead experiments were underway and halting further investment in a non-functional configuration. This dual role (prospective quality gate and diagnostic troubleshooting tool) reflects the practical scope of the computational pre-screening workflow reported in this study.

## 2. Results

This section is divided into in silico and experimental workflows.

### 2.1. In Silico Workflow

#### 2.1.1. Nearest-Neighbour Thermodynamic Analysis

Nearest-neighbour thermodynamic analysis (Figure 1) showed that the capture probe, DNA1, formed a fully complementary, stable duplex with the target and reporter sequences (ΔG = −22.8 kcal/mol, T_m_ = 72.8 °C). However, the capture probe cannot form a stable duplex with the non-target control (ΔG = −4.5 kcal/mol, T_m_ = 6.6 °C), which is below the assay temperature. 3D bound structure models consistent with this confirmed complementarity were generated by molecular docking, as described in Section 2.1.4, rather than by direct geometric construction.

The capture and reporter duplexes and the capture and target duplexes were predicted to have identical stability (ΔG = −22.8 kcal/mol, T_m_ = 72.8 °C). This equal stability reflects sequence identity, not chance. On full sequence confirmation, DNA2, the AuNP-conjugated reporter, 5′-Thiol-MC6-GTCGTGACTGGGTAAACG-3′, and DNA3, the target, 5′-GTCGTGACTGGGTAAACG-3′, were identical 18-nucleotide recognition sequences. They differ only in the 5′ thiol C6 modification required for gold conjugation. In contrast, the non-target sequence shared only a 5-nucleotide complementary stretch with DNA1, predicted to be thermodynamically unstable under assay conditions (ΔG = −4.5 kcal/mol, T_m_ = 6.6 °C), below the 37 °C incubation temperature.

Because DNA2 and DNA3 are sequence-identical, this assay is not a toehold-mediated strand displacement design. There is no sequence asymmetry for the toehold. Instead, displacement is expected to proceed via a concentration-driven mass-action exchange mechanism. A molar excess of unlabelled target added to the solution competes with and progressively displaces the AuNP-labelled reporter from the pre-hybridised DNA1 and DNA2 duplexes. This is driven purely by changes in equilibrium concentrations, not by any difference in binding affinity [7]. This mechanism has a direct precedent in a target-induced strand displacement biosensor previously developed by our group, where the extent of target-to-duplex molar excess was reported to determine the rate and extent of reporter displacement [3]. Under this mechanism, the non-target sequence is correctly predicted to be unable to compete for the DNA1 binding site, as its T_m_ is well below the assay temperature. In contrast, displacement by the target is expected to depend on the molar excess of the target relative to the bead-bound reporter and sufficient equilibration time, rather than on any sequence-level kinetic advantage.

The same computational workflow identified that the capture probe, Bio DNA, of the magnetic bead platform did not complement the AuNP thiol DNA conjugate used in that assay, as described in Section 2.2.2. This provides a structural explanation for the pattern consistent with the non-specific adsorption observed in Section 2.2.2, independent of and prior to any wet lab troubleshooting.

Specifically, Bio DNA, the biotinylated capture probe used to functionalise the streptavidin magnetic beads described in Section 4.2.7, has a single recognition region, GCGGGA, six nucleotides, flanked by a T20 spacer. This region shares at most a 3-nucleotide overlap with the AuNP thiol DNA conjugate used in the same assay, 5′-Thiol-GTCGTGACTGGGTAAACG-3′, which was confirmed to be the same conjugate batch used on both the electrode and bead platforms. This is well below the length needed to form a stable duplex under any of the conditions used, that is, 20 min of pre-hybridisation at 37 °C. One contributing factor worth noting is that the T20 spacer common to both DNA1 and Bio DNA is itself a candidate source of non-specific surface interaction, since poly dT sequences are known to adsorb readily to streptavidin, gold, and other assay surfaces, independent of the recognition region. We have not ruled out its contribution here. This does not change the interpretation above, but we flag it as a further variable for future planned repeats with the corrected Bio DNA sequence.

In contrast, the capture probe used on the electrode platform, DNA1, described in Section 4.2.4, is fully complementary (18 of 18 nucleotides) to the AuNP thiol DNA conjugate, as established above. The capture and reporter pairing of the electrode platform was designed correctly. The sequence mismatch identified here is specific to the capture probe of the magnetic bead platform, Bio DNA.

A corrected Bio DNA sequence for future repeats of the bead-based assay would need to share the proven recognition region of the capture probe, for example, 5′-Biotin-spacer-T20-CGTTTACCCAGTCACGAC-3′, the same 18-nucleotide recognition sequence as the electrode capture probe DNA1, biotinylated in place of its native attachment to the electrode surface. The thermodynamic analysis above already established that this sequence is fully complementary to the AuNP thiol DNA conjugate, ΔG = −22.8 kcal/mol, T_m_ = 72.8 °C.

#### 2.1.2. DNA Secondary Structure Self-Check

As shown in Figure 2, a DNA secondary structure self-check (described in Section 4.1.2) further showed that none of the four assay sequences carried competing intramolecular self-structures strong enough to interfere with their intended intermolecular behaviour. Capture, target, and reporter showed only weak self-hairpins, ΔG = −1.1 and −0.7 kcal/mol, respectively, which were negligible against their −22.8 kcal/mol intermolecular duplex. Non-target and Bio DNA showed no stable self-structure, with a net unfavourable optimal ΔG of +0.3 and +2.5 kcal/mol, respectively. This result is consistent with the conclusions drawn from the nearest-neighbour thermodynamics and wet lab data. According to the criterion set out in Section 4.2.2, this outcome means that molecular dynamics (MD) refinement was not pursued in this study.

Capture, DNA1, and target and reporter DNA2 and DNA3 each showed only a weak, marginally stable self-hairpin, with optimal ΔG values of −1.1 and −0.7 kcal/mol, respectively, located in or near their recognition regions. These values are negligible relative to the −22.8 kcal/mol intermolecular duplex free energy established between these two sequences (Section 2.1.1, Figure 1), a difference of over 20 kcal/mol strongly favouring intermolecular hybridisation over self-folding when both strands are present. In contrast, non-target and Bio DNA showed no stable self-structure. The optimal, most favourable, predicted fold for each was net thermodynamically unfavourable, ΔG = +0.3 and +2.5 kcal/mol, respectively. The mfold web server produced a correspondingly large number of near-degenerate suboptimal structures for both, with Bio DNA in particular producing more than ten structures with similar energy. This is consistent with an unstructured, floppy single strand rather than a sequence with any preferred fold.

These results fully support the conclusions established by the nearest-neighbour thermodynamics and wet lab data. None of the four sequences had a strong enough self-structure to explain the weak or absent target-specific signal observed on either platform. In particular, the absence of any stable fold in Bio DNA rules out self-structure as an alternative explanation for its failure to hybridise with the AuNP thiol DNA conjugate, as described in Section 2.1. This reinforces that this is a genuine sequence complementarity mismatch, rather than a self-folding artefact. Because this check did not reveal any results inconsistent with the wet lab data, we kept MD refinement deprioritised, per the criterion set out below.

#### 2.1.3. Construction of the AuNP-DNA2 Conjugate Model Architecture

To accompany the bioconjugation protocol described in Section 4.2.2, a coarse geometric model illustrating the expected architecture of the AuNP-DNA2 conjugate is provided as Supplementary Figure S1. This is a schematic geometric construction of Fibonacci-distributed thiol attachment points on a 40 nm spherical core, with T20 spacers and recognition segment chain lengths set from standard single-stranded DNA contour length estimates [8]. This is intended to visualise the strand density, spacer, and recognition segment architecture. It is not the output of an MD simulation or an energy minimisation procedure, and we draw no conformational or binding energy claims from it.

#### 2.1.4. Molecular Docking as Corroborative Evidence

As a corroborative and illustrative line of evidence, we used HADDOCK molecular docking to generate structural models for the assay sequence pairs. The scores reported below are indicative and illustrative, not quantitative binding evidence. We used HADDOCK to dock the assay sequences pairwise for both the electrode platform (Method 1) and the magnetic bead platform (Method 2). This platform name is separate from Methods A and B, which refer only to the two immobilisation strategies tested on the electrode platform in Section 4.2.4. We compared complexes expected to represent weak or negative binding (non-target controls) with those expected to represent strong binding and displacement (target conditions). The results of the first RNA-parameterised docking run are summarised in Table 1 and Table 2. The results from the DNA-parameterised rerun are summarised in Section DNA-Parameterised HADDOCK, and Table 3 and Table 4.

The first docking run used HADDOCK’s default RNA parameterisation for both binding partners, as explained in Section 2.1.4. Prior HADDOCK-based aptamer work by this group [9,10] deliberately converted single-stranded DNA aptamer sequences to RNA before docking. This was done because the RNA force fields available in HADDOCK and GROMACS are more thoroughly validated for nucleic acid folding and dynamics than the corresponding DNA parameterisations [11].

However, Section 2.1.1 and Section 2.1.2 already show that DNA1 and DNA2 or DNA3 form a fully complementary standard Watson–Crick DNA duplex with confirmed sequence identity. The structure being docked here is therefore a known, literal DNA-to-DNA pairing, not a folded aptamer of an uncertain shape. Therefore, we repeated all eight docking runs with both chains explicitly typed and parameterised as DNA, using the two-letter nucleic acid residue codes of HADDOCK 2.4, DA, DC, DG, and DT. This departs from the RNA conversion approach used in references [9,10] by design to keep the modelled structure faithful to the actual DNA reagents used in the wet lab assay, as described in Section 4.2.4 and Section 4.2.7. The corrected DNA-parameterised docking results and structures are presented in the DNA-Parameterised HADDOCK subsection.

As shown in Table 2 (RNA-parameterised run), the positive control (Bio DNA with target) scores marginally less favourably than the negative control (−26.5 ± 3.5 vs. −27.0 ± 1.1 HADDOCK score units). This is expected because the Bio DNA recognition region is not complementary to the target sequence, as established in Section 2.1.1. The positive control therefore does not represent a genuinely complementary interaction, and the anomalous score ordering reflects blind docking to a mismatched sequence rather than an experimental artefact.

Table 1 and Table 2, with the corresponding structures shown in Figure 3 and Figure 4, show that the target displacement condition scored most favourably in each case. This agrees with the results of the wet-lab displacement experiments. However, this docking run used the default RNA parameterisation of HADDOCK rather than the explicit DNA residue coding intended for this system, as explained in Section 2.1.4. The RNA folded geometry and thermodynamics of these sequences may differ from those of the DNA duplex used in the assay. Separately, the two platforms also showed opposite control versus displacement ordering. The corrected DNA-parameterised docking results and PyMOL-rendered structures are presented in the DNA-Parameterised HADDOCK subsection below, together with a comparison against these RNA-parameterised results.

##### DNA-Parameterised HADDOCK

To correct the RNA parameterisation used above, we repeated all eight docking runs, four conditions per platform (negative control, positive control, displacement non-target, and displacement target), with both binding partners explicitly typed and parameterised as DNA, using the two-letter residue codes DA, DC, DG, and DT. We followed the same EASY access level HADDOCK 2.4 protocol, active and passive residue convention, and top-ranked cluster selection criterion described in Section 2.1.4. For the displacement conditions, the capture or Bio DNA strand was submitted as the active chain against the incoming target or non-target sequence, with the pre-hybridised reporter strand present but passive, as described in Section 2.1.4.

Comparing the DNA-parameterised results (Table 3 and Table 4, Figure 5 and Figure 6) against the RNA-parameterised results obtained above (Table 1 and Table 2, Figure 3 and Figure 4) shows both a general shift in score size and platform-specific changes in how well the control conditions distinguish target from non-target. Across both platforms, DNA-parameterised HADDOCK scores were substantially more favourable and more negative than the matching RNA-parameterised scores. This reflects the more permissive, better-optimised sampling that HADDOCK provides for true double helical DNA duplexes, compared with the RNA folded geometry that these same sequences would adopt under the default parameterisation.

For Method 1, DNA parameterisation weakened the control condition discrimination relative to the RNA parameterisation result. The negative and positive controls scored almost identically (−63.3 against −62.2, Table 3), compared with a clearer, though still modest, separation under RNA parameterisation (−17.4 against −23.9, Table 1). For Method 2, the opposite occurs. DNA parameterisation sharpens the gap between the Bio DNA negative and positive controls (−41.1 against −25.5, Table 4) relative to the RNA parameterisation result.

Despite this control level variability, the displacement conditions were clearly discriminated under DNA parameterisation for both platforms and in the functionally relevant direction. The displacement target was the single most favourable pose among all eight DNA-parameterised conditions for Method 1 (−72.1, Table 3), and was clearly separated from the displacement non-target (−61.5). For Method 2, the displacement target was likewise its platform’s most favourable pose (−55.7, Table 4), separated from the displacement non-target (−31.2). This mirrors the pattern already noted for the RNA-parameterised data above, where the target displacement condition also scored most favourably in each case. Taken together, the displacement condition result, arguably the more functionally relevant comparison for the actual detection mechanism of this assay, was robust to the choice of nucleic acid parameterisation, even though the control conditions were not.

Taken together, the HADDOCK results in this study function as structural illustrations, as they provide 3D models consistent with the Watson–Crick complementarity established by nearest-neighbour thermodynamics, and they show that target displacement poses are energetically plausible. They are explicitly not presented as independent validations of the displacement mechanism, nor as quantitative binding affinity measurements. Static docking scores cannot establish displacement kinetics, equilibrium constants, or a mechanistic preference for concentration-driven strand exchange. This distinction is reinforced by the parameterisation sensitivity observed: control condition rankings differed between RNA and DNA parameterisations, showing that absolute scores of individual static poses are less reliable than the convergent finding that target displacement is consistently most favourable across all eight runs. Future work should employ dynamic nucleic-acid modelling to complement this static structural picture with kinetic and thermodynamic ensemble information.

### 2.2. Experimental Results

#### 2.2.1. Electrode-Based Displacement Assay, Methods A and B

We obtained preliminary electrochemical current measurements for both electrode-based capture probe immobilisation strategies, as described in Section 4.2.4. These were Method A, electric current-assisted deposition, and Method B, passive overnight incubation under humid conditions. Both methods showed the expected directional current ranking (target > non-target > buffer), but neither comparison reached statistical significance (Method A: *n* = 4–5 independent electrodes, *p* > 0.05 for target vs. non-target; Method B: *n* = 2 electrodes, descriptive only). Replicate numbers were below the minimum of five recommended in the cited literature, and Method A in particular showed substantial between-electrode variability. The full dataset, individual traces, replicate-level values, and statistical comparisons for both methods are provided in the Supplementary Information (Supplementary Note S1, Supplementary Figures S2 and S3). Section 4 discusses the possible contributing factors to this observed variability. Effect sizes (Cohen’s d) were not computed for this dataset owing to insufficient replication; see Supplementary Table S6. All electrode measurements should be regarded as exploratory preliminary observations rather than validation of assay performance.

#### 2.2.2. Magnetic Bead Platform: Computational Design Error Identified and Explained

As shown in Figure 7, at a 1:1.5 dilution, using a 5 µL bead volume, blank-subtracted absorbance at 520 nm, the characteristic AuNP plasmon wavelength was calculated as described below.

In the bead pellet fraction (Figure 7, left), target, non-target, and buffer were of comparable magnitude, 0.411, 0.390, and 0.370 absorbance units, respectively, rather than showing the target-specific decrease expected from successful displacement. In the supernatant fraction (Figure 7, right), the fraction expected to carry the displaced AuNP reporter and therefore the most direct readout of displacement, the target did not exceed non-target or buffer, 0.027, 0.025, and 0.072 absorbance units, respectively. Buffer alone gave the highest absorbance of the three, which was the opposite of the expected pattern. This pattern was observed across the other bead volumes and dilutions tested (see Supplementary Tables S7 and S8). Neither fraction that was expected to show displacement showed the expected pattern.

Subsequent computational sequence analysis (described in Section 2.1) revealed that the biotinylated bead capture probe (Bio DNA) used in this assay did not complement the AuNP thiol DNA conjugate, with a maximum of three nucleotide overlaps. This explains the patterns observed above. The comparable bead pellet absorbance across target, non-target, and buffer conditions is consistent with non-specific AuNP adsorption to the bead surface, rather than sequence-specific pre-hybridisation and target-induced displacement. Non-specific adsorption is not expected to differ between the three conditions.

#### 2.2.3. Bare Electrode Control for Magnetic Bead Pellets

As a negative control for the magnetic bead platform, we tested the pellet fraction remaining after target-induced displacement and magnetic separation, as described in Section 2.2.2 and Section 4.2.7, directly on bare, unmodified electrodes. We used 2 and 5 µL pellet volumes, made up to 100 µL with measuring buffer, matching the main assay for target, non-target, and buffer-only pellet conditions. Across all conditions and both volumes, the current increased without a discrete Faradaic peak, and the target, non-target, and buffer traces were not distinguishable from one another. The full traces are shown in Supplementary Figure S4 and Note S2, and the summary values are shown in Supplementary Table S5. Therefore, the pellet fraction retained no detectable redox signal from the AuNP conjugates after displacement and separation. This is consistent with the AuNP conjugate having already migrated into the supernatant fraction. The UV-Vis absorbance data in Section 2.2.2 (Figure 7) are compatible with that interpretation, but they do not verify it. Those data do not demonstrate target-specific displacement, because the buffer-only condition gave the highest supernatant absorbance of the three conditions tested. This pattern is compatible with redistribution or non-specific release of the AuNP conjugate rather than with target-induced displacement. This observation, together with the UV-Vis results, is why we chose to pursue UV-Vis rather than electrochemical detection for monitoring the distribution of the AuNP conjugate on the magnetic bead platform.

## 3. Discussion

This study compared two DNA1 capture probe immobilisation strategies for an AuNP-amplified target-induced displacement assay. A structural bioinformatics workflow that rationally supports displacement design before and alongside wet-lab testing is also introduced. The results reported so far are preliminary, but the structural bioinformatics component has already shown concrete value in the literature. It identified a specific, correctable design error in the magnetic bead platform before committing further experimental resources. It also independently established that the capture and reporter pairing of the electrode platform was designed correctly.

The computational analyses served two distinct roles in this study, which are important to distinguish. First, the sequence-level screening of the electrode capture and reporter sequences (DNA1, DNA2, DNA3) was performed prospectively (before the electrode experiments were conducted), confirming that the electrode design was thermodynamically sound before resources were committed to wet-lab optimisation. This constitutes the genuine prospective quality-gate function of the pre-screening workflow. Second, the Bio DNA mismatch was identified computationally during the experimental design phase, once the bead assay design had been committed but before the full experimental series was completed. The computational finding halted further investment in the non-functional configuration and provided a mechanistic explanation for the weak, non-differential bead response (a diagnostic troubleshooting role). Both roles are practically valuable, but they represent different points of intervention in the development pipeline, and the manuscript distinguishes them accordingly throughout.

For the magnetic bead platform described in Section 2.2.2, near-identical AuNP absorbance across target, non-target, and buffer conditions was observed at every dilution and bead volume tested. This is best explained by the sequence mismatch identified between the biotinylated capture probe, Bio DNA, and AuNP thiol DNA conjugate (described in Section 2.1), rather than by assay sensitivity limits or displacement kinetics. Since Bio DNA and the conjugate share at most a 3-nucleotide overlap, the 20 min pre-hybridisation step is unlikely to have produced a genuine sequence-specific complex. The AuNP signal retained on the beads was more consistent with nonspecific surface adsorption, which would not be expected to differ between the three test conditions, exactly the pattern observed here. This is a more parsimonious explanation than assay sensitivity, and it points to a specific, actionable correction, a redesigned Bio DNA sequence using the corrected recognition region proposed in Section 2.1.

For the electrode platform (described in Supplementary Note S1), preliminary current measurements showed the expected directional ranking, target > non-target > buffer, for both immobilisation methods. This is consistent with the genuine target-induced displacement, given the confirmed, fully complementary capture and reporter design of this platform. However, the number of replicates was limited. Method A had four to five replicates, and Method B had two. Variability was substantial, particularly for Method A, and neither method reached statistical significance for target versus non-target discrimination. However, for Method A, the comparison between the non-target and buffer yielded a borderline result (*p* = 0.05), with the non-target current elevated relative to the buffer. This is not the pattern expected for a clean specificity control, where the non-target and buffer should behave similarly. We report this result rather than setting it aside, since it runs counter to the specificity pattern supported by the rest of the electrode platform data. With three pairwise tests run for Method A (Bonferroni-corrected threshold α = 0.017, Section 4.2.8), a single borderline result at this significance level does not meet the corrected threshold. Given this variability, the full electrode dataset, traces, replicate-level values, and statistics can be found in the Supplementary Information. The capture and reporter chemistry of this platform remains sound, as independently verified by the sequence-level analysis in Section 2.1. The reproducibility across a larger number of replicates in future studies remains to be established.

Several factors related to the immobilisation chemistry could plausibly contribute to this variability, particularly for Method A. First, the size of the positive potential applied during electric current-assisted deposition may not yet be optimised. A high potential could overpack the capture probe at the electrode surface, whereas a low potential may result in inconsistent surface coverage between replicates. Either of these could introduce steric hindrance that affects subsequent target and reporter binding [12,13]. Second, the capture probe may not be uniformly anchored at its intended attachment point, the 5′ end, adjacent to the polythymine spacer. Non-specific multi-point attachment along the phosphate backbone, rather than single-point anchoring, is a well-documented cause of variable probe orientation and steric hindrance in electrochemical DNA biosensors. This is expected to reduce and destabilise the fraction of capture probes with their recognition region freely accessible for hybridisation [13]. Both factors are consistent with the known immobilisation chemistry for potential-guided DNA deposition, but neither was directly tested here, for example, through surface density or orientation-sensitive characterisation, such as atomic force microscopy (AFM) or quartz crystal microbalance (QCM). These could be plausible contributing factors rather than confirmed mechanisms of action.

Sequence confirmation established that the AuNP-conjugated reporter and target were identical 18-nucleotide recognition sequences. This means that displacement in this system is expected to proceed through concentration-driven mass action exchange rather than toehold-mediated branch migration, as there is no sequence asymmetry available to drive directional invasion. Reconciling this with the assay timescale requires careful consideration. Using the confirmed duplex free energy, ΔG = −22.8 kcal/mol, together with a literature-typical DNA hybridisation association rate constant, k_on_ of approximately 10^6^ M^−1^ s^−1^, the estimated dissociation lifetime of a fully formed capture and reporter duplex at 37 °C is on the order of 10^10^ s (roughly 350 years). This is many orders of magnitude longer than the 30-to-60 min assay incubation. This rules out complete duplex separation and recapture as a plausible displacement pathway on this time scale. Explicitly: K_eq_ = exp(−ΔG°/RT) = exp(22.8/0.616) ≈ 1.17 × 10^16^; k_off_ = k_on_/K_eq_ ≈ 10^6^/(1.17 × 10^16^) ≈ 8 × 10^−11^ s^−1^; τ = 1/k_off_ ≈ 1.25 × 10^10^ s ≈ 397 years (~350 years, rounded). This estimate assumes free-solution conditions and a canonical k_on_ ≈ 10^6^ M^−1^ s^−1^ [14]. It does not account for surface immobilisation, AuNP conjugation, or probe density effects, which are known to alter hybridisation kinetics at surfaces [15,16]. The full derivation with explicit assumptions is provided in Supplementary Note S4.

Therefore, any displacement that occurs is more likely to proceed through transient fraying at the duplex ends, a brief, partial opening of the terminal 1 to 2 base pairs. This occurs on much faster (microsecond to millisecond) timescales, even for otherwise highly stable duplexes, and allows a free target strand to occasionally invade without requiring full separation. This toehold-free, blunt-end displacement pathway has been documented in the DNA nanotechnology literature [7]. It is known to proceed several orders of magnitude more slowly than the genuine toehold-mediated displacement. This is consistent with the weak or absent target-specific signal observed on both the bead (Section 2.2.2) and electrode platforms (Section 2.2.1). This study has practical implications for both platforms. The assay performance under the current design is expected to depend heavily on the molar excess of the target used and on allowing substantially longer equilibration time than is typical for toehold-mediated assays. A genuine sequence-level fix, introducing a deliberate toehold of 6–8 nucleotides between DNA2 and DNA3, would be expected to shift the mechanism to fast, directional, toehold-mediated exchange, rather than relying on this slow fraying-mediated pathway. This mechanism has not been directly demonstrated for the present immobilised, AuNP-conjugated system and should be considered a thermodynamically motivated hypothesis rather than an established conclusion. Concentration-dependent or time-resolved displacement experiments would be required to test this pathway.

The DNA-parameterised HADDOCK rerun, described in Section DNA-Parameterised HADDOCK, adds a further, independent line of support for this mechanistic picture while also tempering how far the docking results as a whole should be trusted. Across both platforms, the displacement target condition remained the single most favourable pose under DNA parameterisation, exactly as it had been under the original RNA-parameterised run and by a wide margin for the electrode platform. The stability of the functionally relevant comparison, target displacement scoring, is the best of all the modelled poses, regardless of how the nucleic acid was parameterised, which is consistent with genuine target-induced displacement being structurally favourable on both platforms. In contrast, the negative and positive control conditions were considerably more sensitive to the choice of parameterisation. Discrimination between them weakened for the electrode platform and reversed for the magnetic bead platform under DNA parameterisation relative to RNA. Because the positive control of the magnetic bead platform is already known (from sequence analysis alone in Section 2.1) and does not represent genuine complementary binding, this reversal is not surprising. However, it does show that the absolute HADDOCK score of a single static pose is a less reliable indicator than the relative ranking of the displacement conditions and that conclusions drawn from control condition scores alone should be treated with more caution than those drawn from the displacement comparison.

## 4. Materials and Methods

### 4.1. In Silico Workflow

#### 4.1.1. Displacement Prediction

To provide a rational, sequence-level basis for the expected displacement behaviour, we predicted the duplex stability between the capture probe DNA1 and each of the reporter probes DNA2, target DNA3, and non-target sequence. We used nearest-neighbour thermodynamic modelling with the Santa Lucia unified parameter set [17]. We used the same monovalent salt concentration and Python (version 3.11) implementation of this parameter set as in a previous study by our group [1,3]. This predicted the Gibbs free energy (ΔG) at 37 °C and the melting temperature (T_m_) at a 10 µM strand concentration for each duplex. The non-target sequence used for this analysis and for the matching wet-lab specificity control was the linear target DNA sequence from a previously published assay targeting HEV, developed by this group [3]. This sequence was chosen because it is a well-characterised sequence unrelated to the negative control.

#### 4.1.2. DNA Secondary Structure Self-Check

Each of the five assay sequences, capture, target, reporter, non-target, and Bio DNA, was checked individually for competing intramolecular secondary structures. We used the mfold web server DNA folding form (https://www.unafold.org/DNA_form.php, accessed on 14 July 2026) in the DNA mode at 37 °C, matching the assay conditions. This follows the same recommendation made in the Liu and Lu [18] protocol for DNA probe sequence optimisation.

#### 4.1.3. Construction of AuNP-DNA2 Model Architecture

In addition to the bioconjugation protocol described in Section 4.2.2, we constructed a coarse geometric model. This model used Fibonacci-distributed thiol attachment points on a 40 nm spherical core.

#### 4.1.4. HADDOCK-Based Docking Method

Nearest-neighbour thermodynamics (Section 4.1.1) and DNA secondary structure self-check (Section 4.1.2) together provide a sequence-level basis for the intended displacement mechanism directly from base pairing rules. On their own, these two analyses are sufficient to support the conclusions regarding sequence complementarity drawn in this study. To generate corroborative, illustrative three-dimensional (3D) bound structure models for visual presentation in Section 2.1.4, we performed pairwise molecular docking using HADDOCK version 2.4 [11]. We chose docking over direct geometric construction from canonical helical parameters because a static illustrative geometry would not address the docking-based structural comparison presented in Section 2.1.4 and DNA-Parameterised HADDOCK.

We performed four docking runs on each platform, for a total of eight runs. These covered four structurally distinct capture and partner pairings for each platform. These were a negative control (capture with non-target), a positive control (capture with target), and two conditions representing the AuNP conjugate partner strand under non-target and target displacement, called Displacement Non-Target, and Displacement Target, respectively. We applied this structure to Method 1, the electrode platform, where the capture probe DNA1 was paired with the non-target sequence, target and reporter sequence DNA2 and DNA3, and matching displacement conditions. We also applied Method 2, the magnetic bead platform, where Bio DNA was paired with the same four conditions.

We first ran all eight docking jobs at the EASY access level of the HADDOCK 2.4 web server, with all settings left on default. This follows the protocol described in a prior aptamer docking study by this group [9]. Because the default setting type specifies nucleic acid chains as RNA rather than DNA, this first set of runs used HADDOCK’s default RNA parameterisation for both binding partners, even though the actual assay reagents were DNA. Section 2.1.1 and Section 2.1.2 already show, from nearest-neighbour thermodynamics and the DNA secondary structure self-check, that DNA1 and DNA2 or DNA3 form a fully complementary, standard Watson–Crick DNA duplex with confirmed sequence identity. The structure being docked is a known, literal DNA-to-DNA pairing, not a folded aptamer of an uncertain shape. Therefore, we repeated all eight docking runs with both chains explicitly typed and parameterised as DNA. We used the two-letter nucleic acid residue codes of HADDOCK 2.4, DA, DC, DG, and DT, which the web server supports alongside its one-letter RNA style default. This DNA-parameterised rerun is different from the RNA conversion approach used in references [9,10] by design. The aim was to keep the modelled structure in line with the actual DNA reagents used in the wet lab assay, as described in Section 4.2.4 and Section 4.2.7. Both the original RNA-parameterised results and the DNA-parameterised results are reported in Section 2.1.4 and Section DNA-Parameterised HADDOCK, respectively, so that both are available for comparison.

Following the active and passive residue convention used in reference [9], where interface residues on the larger binding partner are set as active and the entire smaller partner is treated as interacting, we set the full recognition region of each capture strand, DNA1, or Bio DNA, as active. We treated the full complementary region of the matching partner strand, DNA2 or DNA3, or the non-target sequence as the interacting region. This reflects the short length and full or partial complementarity of the oligonucleotides. For the Displacement Non-Target and Displacement Target conditions, the HADDOCK web interface only accepts one chain of a pre-formed complex as the active docking partner. Therefore, the capture strand or Bio DNA strand, chain A, was submitted as the active partner against the incoming target or non-target sequence, with the pre-hybridised reporter strand present in the same input structure as chain B, passive. This models the competitive displacement of the reporter by the incoming strand, matching the mass action displacement mechanism described in Section 2.1.1, rather than a simultaneous three-body docking, which the server does not support. Consequently, the docking results represent pairwise structural assessments of binding compatibility, which are structural illustrations, not mechanistic proofs of displacement kinetics or equilibrium.

Clustering was performed using the default HADDOCK 2.4 method, with a maximum of 200 models considered for clustering and the top 10 clusters generated. We selected the top-ranked cluster, which had the most favourable mean HADDOCK score and correspondingly the most negative Z-score, as the representative structure for each condition. This follows the same convention used in previous studies [9,10]. Because each docking job generates its own separate set of clusters, the Z-score of a condition is only meaningful relative to the other clusters within that same job. Z-scores should not be compared across different conditions or tables. Only the raw HADDOCK score was used for comparisons across conditions. Sampling at each docking stage, rigid-body docking, semi-flexible refinement, and water refinement were performed using the HADDOCK 2.4 default settings, following the same precedent. The root mean square deviation (RMSD) value (reported in Table 1, Table 2, Table 3 and Table 4) is the HADDOCK server standard distance of the representative structure from the single overall lowest-scoring model generated across all clusters in that run. It is not a measure of how tightly the representative cluster’s members agree with each other, and a large value does not, by itself, indicate poor docking quality.

For visual presentation, we rendered the lowest energy structure from the top-ranked cluster of each condition using PyMOL, version 2.5, Schrödinger LLC. This is the same visualisation software used in a previous study [11]. We used ray tracing at a 3600-pixel resolution for publication-quality figures.

### 4.2. Experimental Workflow

All experimental work was carried out at the Sensor Technology Laboratory, Pilot Plant Development and Training Institute, King Mongkut’s University of Technology Thonburi (KMUTT), Bang Khun Thian Campus, Bangkok, Thailand, and all the reagents were sourced accordingly. Commercial, citrate-stabilised 40 nm AuNPs, optical density (OD) 1, DCN Diagnostics, Carlsbad, CA, USA, tris(2-carboxyethyl) phosphine (TCEP), Tris acetate buffer, bovine serum albumin (BSA), Tween 20, phosphate-buffered saline (PBS), and potassium chloride were used as received unless stated otherwise. The reagents and their suppliers correspond to the materials used in prior publications from the group [1,3].

Dynabeads M-280 Streptavidin (Thermo Fisher Scientific (Waltham, MA, USA), Lot 111199571) was used for the magnetic bead-based capture strategy described in Section 4.2.7.

Potassium chloride, sodium, and potassium phosphate salts for PBS, Tween 20, TCEP, Tris base, and acetic acid for Tris acetate buffer were obtained from Sigma-Aldrich, St. Louis, MO, USA [1,3]. Screen-printed carbon electrodes (SPCE) were obtained from Quasense, Bangkok, Thailand [1,3]. All custom oligonucleotides, DNA1, DNA2, DNA3, and Bio DNA, were obtained from Integrated DNA Technologies (IDT), Coralville, IA, USA [1,3]. The potentiostat used was a Metrohm Autolab (Utrecht, The Netherlands) controlled by General Purpose Electrochemical System (GPES) software, version 4.9, [1,3]. The source of the AuNP stock is given above and is further detailed in Section 4.2.1. BSA, used for electrode blocking in Section 4.2.4, was purchased from Sigma-Aldrich (USA).

#### 4.2.1. Sources and Preparation of AuNPs

Commercial citrate-stabilised 40 nm AuNPs at OD 1 were purchased from DCN Diagnostics (USA). This corresponds to the AuNP source reported in a previous study [3]. We also attempted an in-house citrate reduction synthesis of AuNPs following the protocol of Turcu Stiolica et al. [19]. However, the AuNPs that were conjugated in Section 4.2.2 and characterised in Section 4.2.3, and that generated the results reported in this study, were the commercial DCN Diagnostics stock described above, not the in-house synthesised particles.

All AuNP preparations and downstream conjugation work described in this study were carried out at the KMUTT Bang Khun Thian Campus, Bangkok, Thailand.

#### 4.2.2. Bioconjugation of AuNPs with the Thiolated Reporter Probe DNA2

Following the Liu and Lu [18] protocol for DNA probe sequence optimisation, 3 µL of disulfide-deprotected DNA2 was used per 1 mL AuNP aliquot. Three aliquots were conjugated in parallel, so 9 µL of DNA2 was used in total across the full preparation. Then, 500 mM Tris acetate buffer (pH 8.2) was added dropwise with gentle mixing to a final concentration of 5 mM. This was followed by the dropwise addition of 1 M sodium chloride to a final concentration of approximately 0.1 M. The mixture was stored overnight before use. This step progressively increased the ionic strength of the solution. This screens the electrostatic repulsion between DNA-functionalised AuNPs, which stabilises the conjugate against aggregation, while adding the salt slowly minimises premature aggregation.

On the third day, the three conjugate tubes were centrifuged at 16,000× *g* at 4 °C, and the supernatant, which contained unbound DNA2, was removed. The pellets were resuspended in a wash buffer containing 100 mM sodium chloride and 25 mM Tris acetate at pH 8.2, centrifuged again at 16,000× *g* at 4 °C, and the supernatant was discarded to remove residual free oligonucleotides. The conjugates were then combined and resuspended in a 1 mL buffer containing 300 mM sodium chloride and 25 mM Tris acetate at pH 8.2, and stored at 4 °C, protected from light, until use.

#### 4.2.3. Physicochemical Characterisation of AuNPs

For the commercial AuNP stock described in Section 4.2.1, we relied on the manufacturer’s specifications for particle identity, size, and colloidal stability rather than independently repeating this characterisation. The extinction coefficient-based method of Haiss et al. [20] describes how ultraviolet-visible spectrophotometry (UV-Vis) and dynamic light scattering (DLS) can confirm the AuNP identity and estimate the nanoparticle concentration. This is the basis for the manufacturer specifications we relied on.

We did not independently characterise the AuNP-DNA2 bioconjugate, as described in Section 6.

#### 4.2.4. Electrode Preparation and Capture Probe DNA1 Immobilisation

The capture probe DNA1, sequence 5′-TTTTTTTTTTTTTTTTTTTT-CGTTTACCCAGTCACGAC-3′, comprised a 20-thymine spacer (T20) and an 18-nucleotide target-complementary recognition region. This design was the same as that used previously by our group [1,3]. DNA1 was immobilised on carbon working electrodes (SPCE, Quasense, Thailand) [1] using two independent strategies. These were evaluated in parallel to compare their performances.

##### Method A: Electric Current Assisted Immobilisation

The protective plastic covers were removed from the four carbon electrodes. The electrodes were washed three times with ultrapure water and dried under a stream of nitrogen gas. A 100 µL aliquot of DNA1 (10 µM) was applied to each electrode. An induced positive surface charge was used to electrostatically drive the attachment of DNA1 through its negatively charged phosphate backbones. After successful current-driven immobilisation, the electrodes were washed and dried under nitrogen gas, blocked with 0.1% BSA, dried under nitrogen, washed with 0.1% Tween 20 in 1× PBS, and dried again under nitrogen.

##### Method B: Passive Overnight Immobilisation

Washed and dried carbon electrodes were incubated with 10 µL of the same DNA1 stock (10 µM) overnight in a humid environment at 4 °C. The next day, the electrodes were processed in the same manner as in Method A, from the washing step. The electrodes were washed, dried under nitrogen, blocked with 0.1% BSA, dried, washed with 0.1% Tween 20 in 1× PBS, and dried under nitrogen.

#### 4.2.5. Reporter Probe Hybridisation and Baseline Measurement

For both immobilisation methods, the electrodes were exposed to the AuNP-conjugated reporter probe DNA2. In Method A, the reporter conjugate was applied directly after blocking and washing. In Method B, the reporter conjugate was hybridised to pre-immobilised DNA1 for 30 min to 1 h. In both cases, the electrodes were washed with 1× phosphate-buffered saline (PBS) and dried under nitrogen gas.

A 100 µL aliquot of the measuring buffer, 0.1 M phosphate, and 1 M potassium chloride was applied to each electrode. Baseline electrochemical readings were recorded using a potentiostat (Metrohm Autolab, The Netherlands) controlled by GPES software. This equipment is the same as that reported in previous studies [1,3].

#### 4.2.6. Target Induced Displacement Assay and Final Measurement

The electrodes were dried under nitrogen gas. A 5 µL aliquot of the target solution, DNA3, or the matching non-target or buffer control was added to 95 µL of the measuring buffer directly on the electrode surface. Final electrochemical readings were recorded using the same instrument settings. Three conditions were evaluated in parallel, each with four to five electrode replicates to ensure reproducibility. Section 2.2.1 provides the exact counts for each condition. The conditions were target DNA (DNA3), non-target DNA (the HEV linear sequence, as a sequence-unrelated negative control), and measuring buffer alone as a blank control.

The non-target control sequence was 5′-CCGGCGGTGGTTTCTGGGGTGAC-3′. This is the linear target DNA from a previously published hepatitis E virus (HEV) assay by this group [3], and it was chosen as a sequence unrelated to the negative control. Section 2.1 provides computational confirmation that it does not complement DNA1.

The displacement of the AuNP-conjugated reporter probe by the target sequence was expected to produce a measurable increase in the current relative to the non-target and buffer-only controls. This is consistent with the concentration-driven displacement mechanism described in Section 2.1.

#### 4.2.7. Alternative Capture Strategy Using Streptavidin Magnetic Beads

As an alternative to surface immobilisation on carbon electrodes, we developed a magnetic bead-based capture strategy using Dynabeads M-280 Streptavidin (Thermo Fisher Scientific). We followed the manufacturer-recommended nucleic acid immobilisation protocol [21].

A 100 µL aliquot of Dynabeads M-280 Streptavidin suspension was placed on a magnetic stand and the supernatant was discarded. The beads were washed three times by resuspension in 1× Binding and Washing (B&W) buffer. This was prepared by diluting 2× B&W buffer, 10 mM Tris HCl at pH 7.5, 1 mM EDTA, 2 M sodium chloride, diluted with distilled water at a 1:1 ratio. The washed beads were resuspended in 200 µL of 2× B&W buffer and mixed with 4 µL of biotinylated capture DNA (Bio DNA), sequence 5′-Biotin-spacer-T20-GCGGGA-3′, and 196 µL of ultrapure water. This brought the sodium chloride concentration to 1 M for optimal binding to occur. The mixture was incubated for 10 min at room temperature with gentle rotation, following the manufacturer’s recommendation that oligonucleotides under 30 bases require a maximum of 10 min incubation [12]. The binding efficiency was assessed by comparing the biotinylated DNA concentration before and after coupling, as measured using a NanoDrop One Microvolume UV-Vis Spectrophotometer (Thermo Fisher Scientific, Waltham, MA, USA). Bio DNA-coated beads were stored at 4 °C until use.

For the displacement assay, aliquots of Bio DNA-coated beads (2 or 5 µL) were combined with the AuNP thiol DNA conjugate (8 or 5 µL, respectively), so that the bead and conjugate volumes summed to 10 µL in every tube, and made up to 30 µL with 1× phosphate-buffered saline (PBS). Tubes were incubated for 20 min at 37 °C to allow pre-hybridisation, and then placed on a magnetic rack. The unbound fraction was removed and retained for further analysis. The pellet was washed three times with 1× PBS at pH 7.4 and resuspended in 50 µL of 1× PBS. Target DNA, non-target DNA, or measuring buffer (30 µL each) was then added to give a final volume of 80 µL per tube, and the tubes were incubated for a further 30 min at 37 °C. The tubes were then returned to the magnetic rack. The supernatant, which contained any displaced AuNP conjugates, was removed and retained separately from the pellet, which was resuspended in an equal volume of ultrapure water. All fractions, the unbound fraction, the supernatant, and the resuspended pellet, were photographed at each stage and analysed by UV-Vis absorbance spectroscopy (400–800 nm), as described in Section 2.2.2. Four tube replicates were prepared for each target, non-target, and buffer condition.

#### 4.2.8. Statistical Analysis

The nearest-neighbour duplex thermodynamic parameters, ΔG and T_m_, were calculated using the Santa Lucia unified parameter set [17]. To compare the electrochemical current response between the target, non-target, and buffer conditions described in Section 2.2.1, we used a two-tailed Welch *t*-test, which assumes unequal variances, with significance set at *p* < 0.05. This test was chosen over Student’s *t*-test because the replicate variances differed between conditions. All UV-Vis absorbance data, described in Section 2.2.2, were reported as blank-subtracted single-well measurements. We did not perform a formal statistical comparison for this dataset because it had only one replicate per condition. See the limitations noted in Section 6 for details. It is worth noting that the threshold *p* < 0.05 stated above applies before multiple-comparison correction. After Bonferroni correction for three Method A comparisons, the corrected threshold is α = 0.017 (see paragraph below).

Formal pairwise comparisons were performed for Method A only, which had four to five independent electrode replicates per condition. Three pairwise Welch *t*-tests were run: target vs. non-target, target vs. buffer, and non-target vs. buffer. A Bonferroni correction was applied to these three comparisons (α_corrected_ = 0.05/3 = 0.017). All three Method A comparisons remained non-significant after correction. Independent replicates are defined as independently prepared electrodes from separate experimental sessions. Effect sizes (Cohen’s d) were not computed; see Supplementary Table S6.

Method B (*n* = 2 independent electrodes per condition) was not subjected to formal inferential testing. With only two replicates, no Welch *t*-test or equivalent parametric test can provide reliable inference. A minimum of *n* = 5 independent replicates per condition is required for meaningful statistical power in this context. Method B current measurements (target: 1.96 µA; non-target: 1.01 µA; buffer: 0.28 µA) are therefore reported as descriptive observations only and should not be interpreted as statistically validated findings. They are consistent with, but do not independently confirm, the directional trend observed in Method A.

## 5. Conclusions

This study evaluated an in silico pre-screening workflow as a design-assessment tool for AuNP-amplified DNA displacement assays, applied as a proof-of-concept case study across two electrode immobilisation strategies and a magnetic bead platform. The computational workflow, integrating nearest-neighbour thermodynamics, DNA secondary-structure analysis, and molecular docking, evaluated the sequence-level and structural basis of the intended displacement mechanism before and alongside experimental testing. Sequence analysis confirmed 18-nucleotide Watson–Crick complementarity among the relevant electrode-platform probes and identified a specific mismatch in the magnetic bead capture probe, providing a plausible explanation for the weak, non-differential response observed experimentally and enabling a corrected sequence to be proposed for future experimental validation. The corrected Bio DNA sequence (5′-Biotin-spacer-T20-CGTTTACCCAGTCACGAC-3′) has not been experimentally tested in the present study and remains a computational prediction requiring synthesis and bead assay validation. Molecular docking using both RNA and DNA parameterisations consistently favoured target over non-target displacement on both platforms, whereas the corresponding control comparisons were more sensitive to the nucleic acid parameterisation and should therefore be interpreted cautiously. Preliminary electrochemical measurements showed the anticipated directional trend for target-induced displacement, although differences were not statistically significant at the replicate numbers tested. Collectively, these findings indicate that the correctly designed electrode-based configuration provides a suitable basis for further optimisation, whereas the analytical potential of the magnetic bead configuration remains to be evaluated once the capture sequence has been corrected. Because the magnetic bead configuration contained a sequence mismatch, the present data do not permit a fair comparative assessment of the intrinsic potential of the two configurations. These findings also demonstrate the practical value of computational pre-screening in biosensor development. Integrating sequence thermodynamics and structural modelling with experimental testing can identify correctable design limitations, interrogate proposed displacement mechanisms, and guide subsequent optimisation before additional experimental resources are committed.

## 6. Limitations and Future Perspectives

Several limitations should be considered when interpreting the findings of this study. First, the physicochemical properties of the commercial AuNP stock were based on the manufacturer’s specifications rather than independently re-verified for the experimental batch. UV-Vis spectroscopy, dynamic light scattering (DLS), transmission electron microscopy (TEM), and scanning electron microscopy (SEM) were not independently performed to characterise the AuNP stock or the AuNP-DNA2 bioconjugate. Future studies should therefore include physicochemical characterisation of both the AuNP stock and resulting bioconjugate to confirm particle size, dispersion, morphology, and conjugation-associated changes.

Second, the electrode-based experimental dataset was limited in replication. Each replicate represented an independently prepared electrode, with four to five replicates for Method A and two replicates for Method B per condition (target, non-target, and buffer). Although the measurements showed the anticipated directional response, these replicate numbers were insufficient to establish statistically robust differences between the immobilisation strategies, particularly given the variability observed for Method A. Accordingly, the complete electrode dataset is provided in Supplementary Note S1, and additional independent replicates will be required before definitive analytical comparisons between the two immobilisation methods can be made. Potential contributors to the observed variability, including the applied potential during current-assisted deposition and capture-probe orientation or multi-point attachment, are discussed in Section 3. The magnetic bead platform was additionally affected by the sequence mismatch identified computationally in Section 2.1. Consequently, these data should not be interpreted as a definitive assessment of target-induced displacement performance. Rather, they characterise the behaviour of the mismatched configuration under the tested conditions and demonstrate the practical value of sequence-level computational pre-screening for identifying correctable assay-design limitations. Re-synthesis of the Bio-DNA capture probe using the corrected recognition sequence proposed in Section 2.1, followed by adequately replicated testing, will be required to evaluate the analytical performance of the corrected magnetic bead platform. The electrode-based platform was not affected by this sequence-design limitation because its capture and reporter sequences exhibited the intended complementarity.

Third, the AuNP-DNA2 conjugate architecture presented in Supplementary Figure S1 is a coarse-grained illustrative representation consistent with the confirmed sequence complementarity and should not be interpreted as an energy-minimised or experimentally resolved structure. The three-dimensional capture-target and capture-reporter complexes were generated independently using HADDOCK under both the default RNA and explicit DNA parameterisations. Although target displacement was consistently favoured over non-target displacement under both parameterisations, the corresponding control comparisons were more sensitive to the choice of nucleic-acid parameterisation. The docking results should therefore be interpreted as corroborative structural evidence supporting the proposed displacement mechanism rather than as quantitative estimates of binding affinity or definitive structural validation. Molecular dynamics simulations were not performed and represent an opportunity for future assessment of the stability and conformational behaviour of the docked complexes.

Fourth, direct electrochemical measurements of the magnetic bead pellets did not yield distinguishable target, non-target, or buffer signals. Together with the corresponding UV-Vis observations, these findings were compatible with the interpretation that the AuNP conjugate was predominantly present in the supernatant fraction following the displacement procedure, and they informed the use of UV-Vis detection for monitoring the distribution of the AuNP conjugate on the magnetic bead platform. The supernatant UV-Vis data do not establish target-specific displacement, and the buffer-only condition gave the highest supernatant absorbance of the conditions reported. However, because the bead capture sequence requires correction and subsequent experimental reassessment, these observations should be interpreted within the constraints of the current platform configuration. All prepared electrode and tube replicates were retained in the reported analyses, and no replicates were excluded.

Fifth, the nearest-neighbour thermodynamic analysis and DNA secondary-structure assessment are sequence-level quality gates; they cannot capture interfacial effects such as steric constraints, surface confinement, changes in the local ionic environment at the SPCE/solution interface, or probe density effects. All these factors are known to alter hybridisation kinetics relative to free-solution predictions [15,16]. The secondary-structure analysis also evaluates individual strands in isolation and does not capture intermolecular folding or competitive hybridisation effects. These limitations are inherent to the sequence-level tools and are acknowledged explicitly here.

Sixth, the exploratory electrode and bead measurements lacked dedicated positive controls (known AuNP concentration at electrode surface) and negative controls (electrode or bead assay without AuNP-DNA2). Their absence means that specific reporter release cannot be rigorously distinguished from non-specific AuNP adsorption or detachment. Future experiments should include a full set of positive and negative controls to establish assay specificity unambiguously.

Future work should prioritise: (1) synthesis and adequately replicated bead assay testing of the proposed corrected Bio DNA sequence (5′-Biotin-spacer-T_20_-CGTTTACCCAGTCACGAC-3′); (2) introduction of a deliberate toehold domain of 6–8 nucleotides to shift the displacement mechanism from slow concentration-driven exchange to fast, directional toehold-mediated branch migration; (3) increasing electrode replicates to *n* ≥ 5 per condition for both immobilisation strategies; (4) independent characterisation of the AuNP stock and AuNP-DNA2 conjugate by TEM, DLS, and agarose gel electrophoresis; and (5) dynamic nucleic-acid modelling (e.g., oxDNA) to complement the static HADDOCK with kinetic and thermodynamic ensemble information.

## Figures and Tables

**Figure 1:**
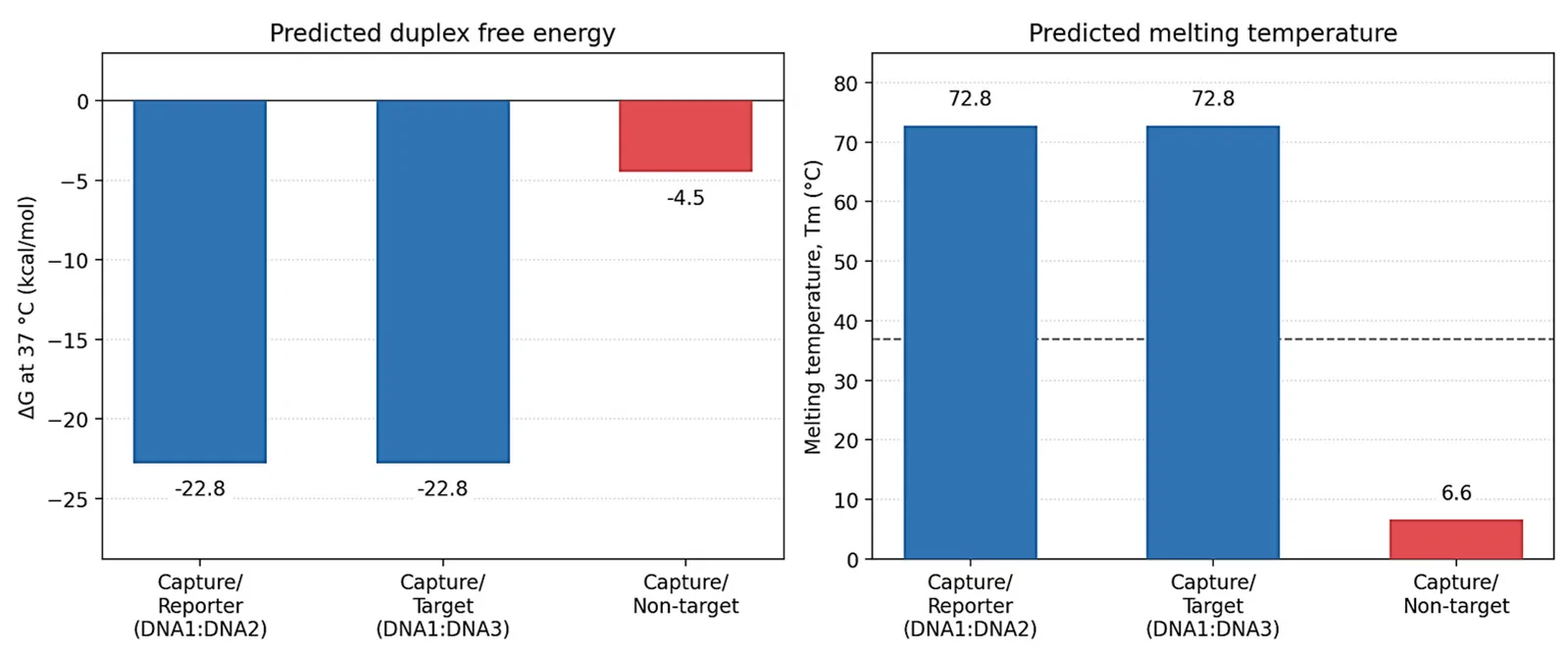
Predicted duplex stability (ΔG at 37 °C, (**left**)) and melting temperature (T_m_, (**right**)) for the capture probe (DNA1) paired with the gold nanoparticle (AuNP) reporter (DNA2), the target (DNA3), and the non-target sequence, calculated by nearest neighbour thermodynamics.

**Figure 2:**
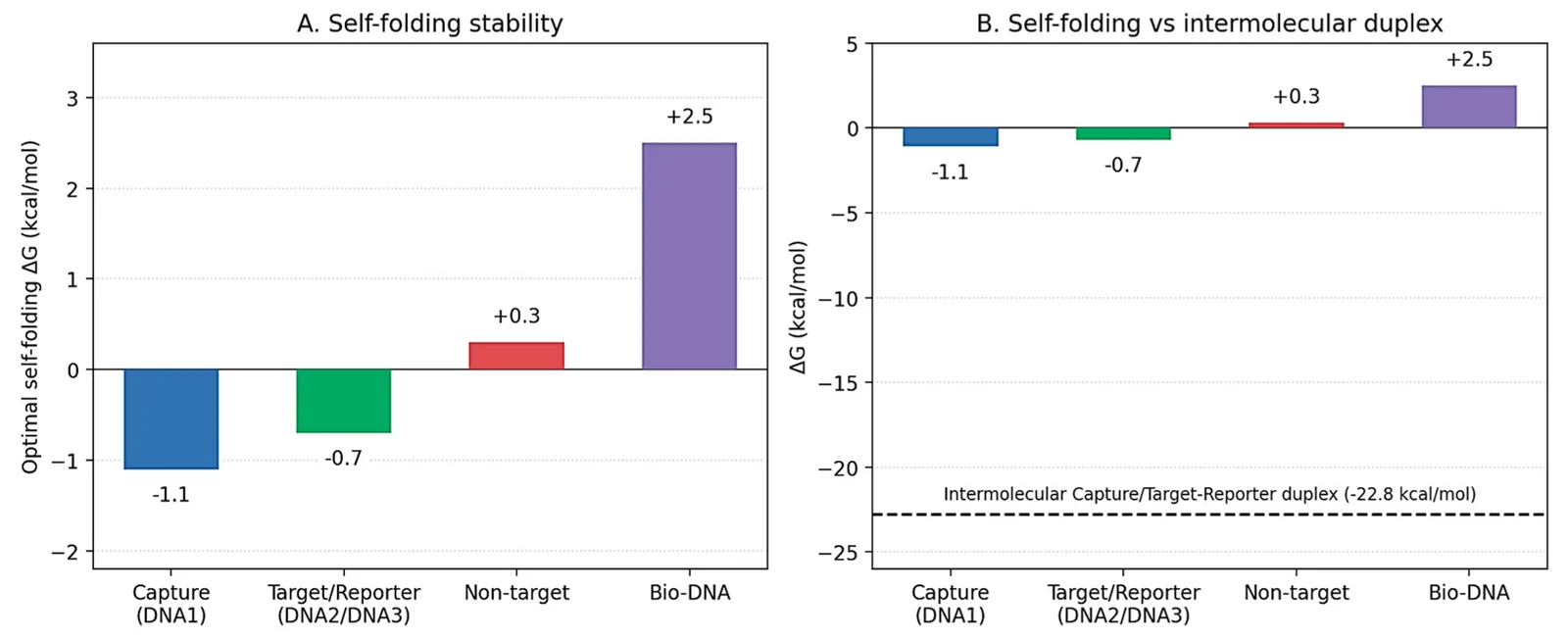
DNA secondary structure self-check, mfold, DNA mode, 37 °C. Panel (**A**) shows the optimal, minimum, self-folding ΔG for each of the four sequences. Panel (**B**) shows the same self-folding values alongside the previously established intermolecular duplex free energies (Figure 1). This shows that self-structure is negligible relative to the intended intermolecular interactions.

**Figure 3:**
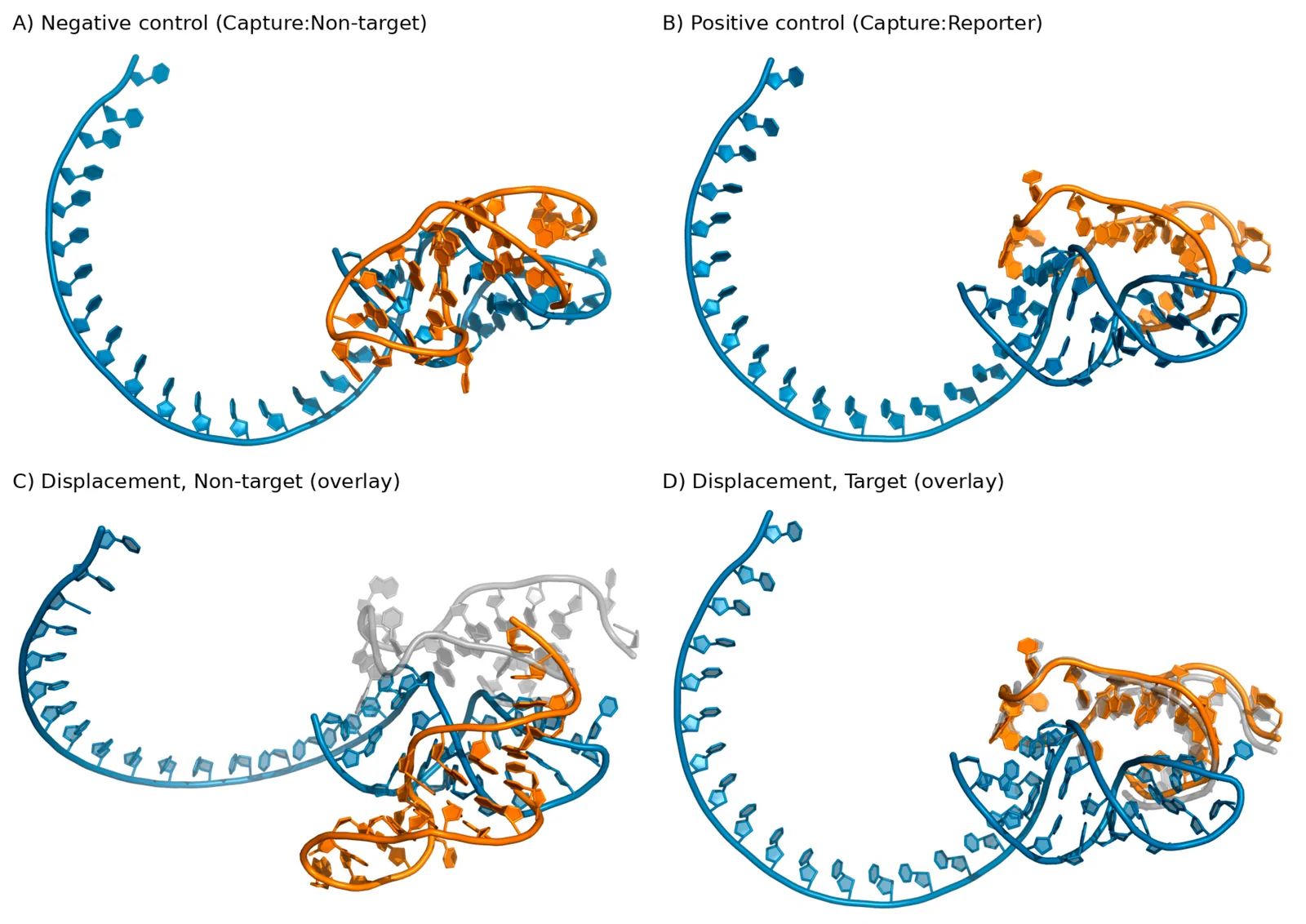
PyMOL rendered lowest energy HADDOCK-docked structures for the electrode platform, Method 1 (RNA parameterised). (**A**) negative control (capture with non-target). (**B**) positive control (capture with reporter/target). (**C**) displacement (non-target). (**D**) displacement (target). The capture strand, DNA1, is shown in blue. The target, non-target, or reporter (target) strand is shown in orange throughout. In panels (**C**,**D**), the displaced reporter strand is also shown, in transparent grey, to make the displacement event visible. HADDOCK does not dock three strands at once, so this reporter position was not solved jointly with the incoming target or non-target strand. It is a structural overlay, the reporter bound pose from panel (**B**), superimposed onto the capture strand of the displacement pose using the shared capture strand as the alignment reference (RMSD below 0.7 Å). Any overlap between the grey reporter and the orange incoming strand reflects them competing for the same binding site on the capture strand.

**Figure 4:**
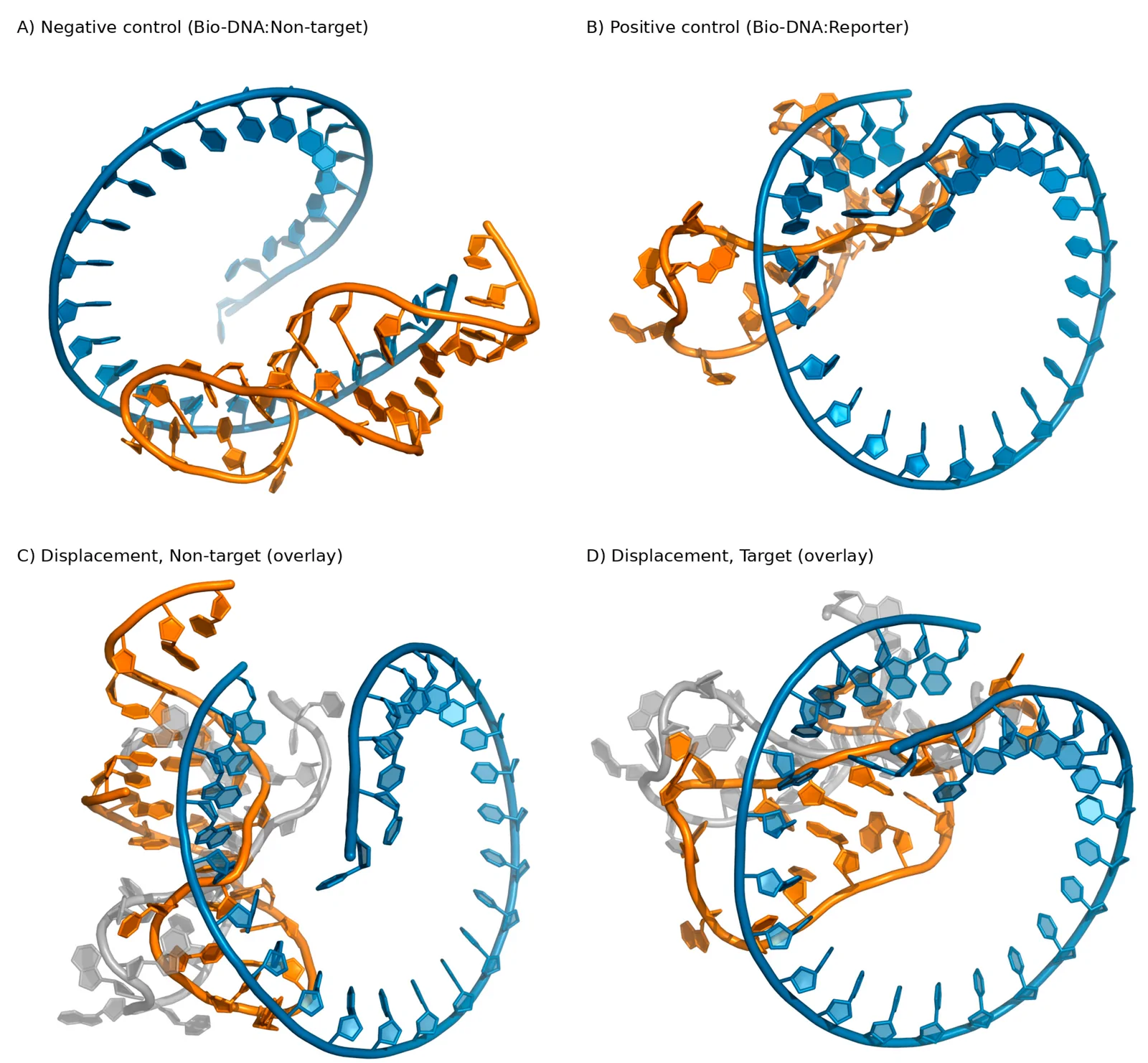
PyMOL rendered lowest energy HADDOCK-docked structures for the magnetic bead platform, Method 2, RNA parameterised. (**A**) negative control (Bio DNA with non-target). (**B**) positive control (Bio DNA with reporter/target). (**C**) displacement (non-target). (**D**) displacement (target). The capture strand (Bio DNA) is shown in blue. The target, non-target, or reporter strand is shown in orange throughout. Panel (**B**) does not represent a genuinely complementary duplex. The actual recognition region of Bio DNA is not complementary to the target sequence, described in Section 2.1. This pose reflects blind docking rather than confirmed sequence-specific binding. In panels (**C**,**D**), the displaced reporter strand is also shown, in transparent grey, to make the displacement event visible. HADDOCK does not dock three strands at once, so this reporter position was not solved jointly with the incoming target or non-target strand. It is a structural overlay, the reporter bound pose from panel (**B**), superimposed onto the capture strand of the displacement pose using the shared capture strand as the alignment reference (RMSD below 0.7 Å). Any overlap between the grey reporter and the orange incoming strand reflects them competing for the same binding site on the capture strand.

**Figure 5:**
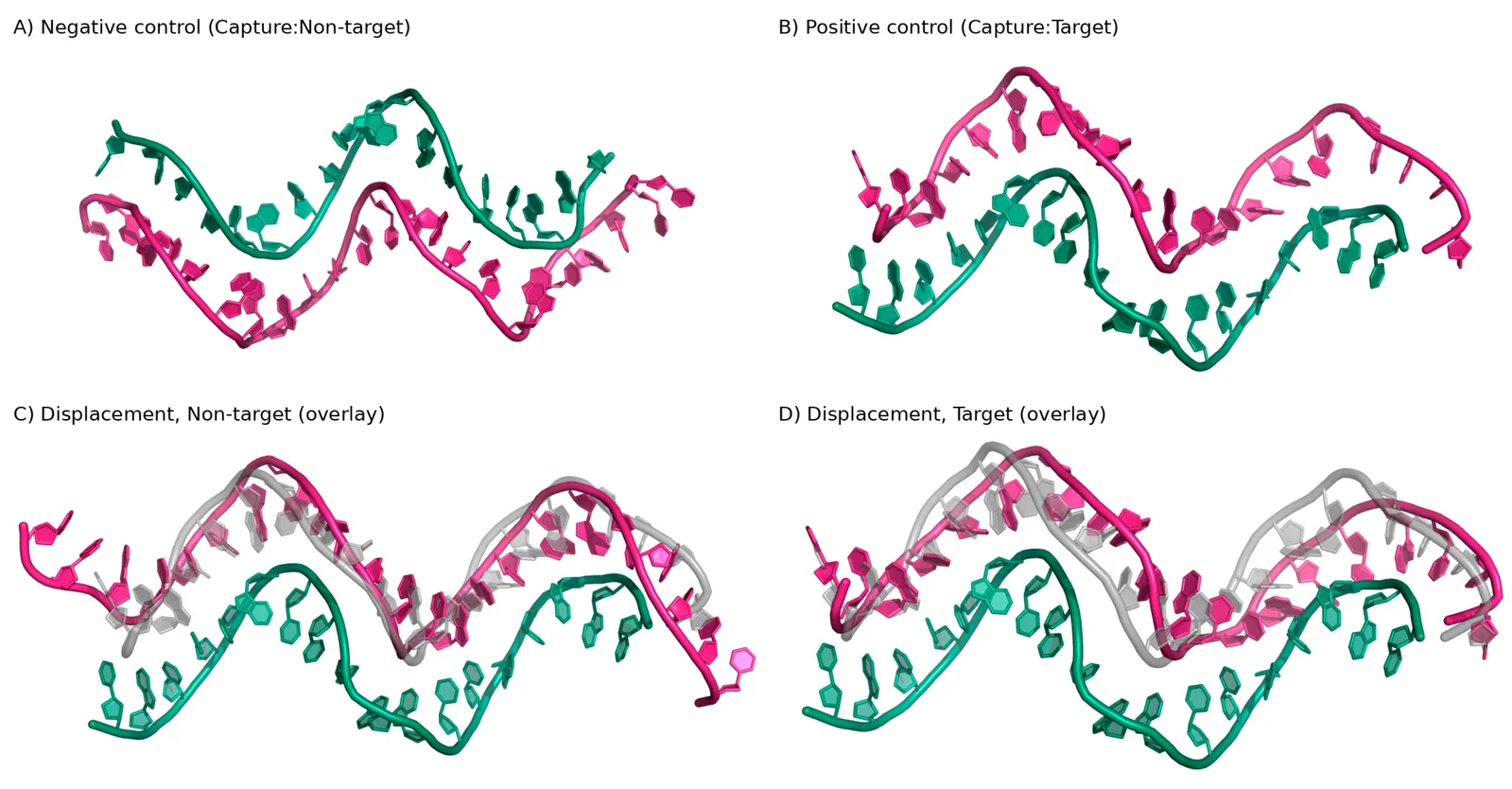
PyMOL rendered lowest energy DNA-parameterised HADDOCK-docked structures for the electrode platform, Method 1. (**A**) negative control (capture with non-target). (**B**) positive control (capture with target). (**C**) displacement (non-target). (**D**) displacement (target). The capture strand, DNA1, is shown in teal. The target or non-target strand is shown in magenta. In panels (**C**,**D**), the displaced reporter strand is also shown, in transparent grey, to make the displacement event visible. HADDOCK does not dock three strands at once, so this reporter position was not solved jointly with the incoming target or non-target strand. It is a structural overlay, the reporter bound pose from panel (**B**), superimposed onto the capture strand of the displacement pose using the shared capture strand as the alignment reference (RMSD below 1.1 Å). Any overlap between the grey reporter and the magenta incoming strand reflects them competing for the same binding site on the capture strand.

**Figure 6:**
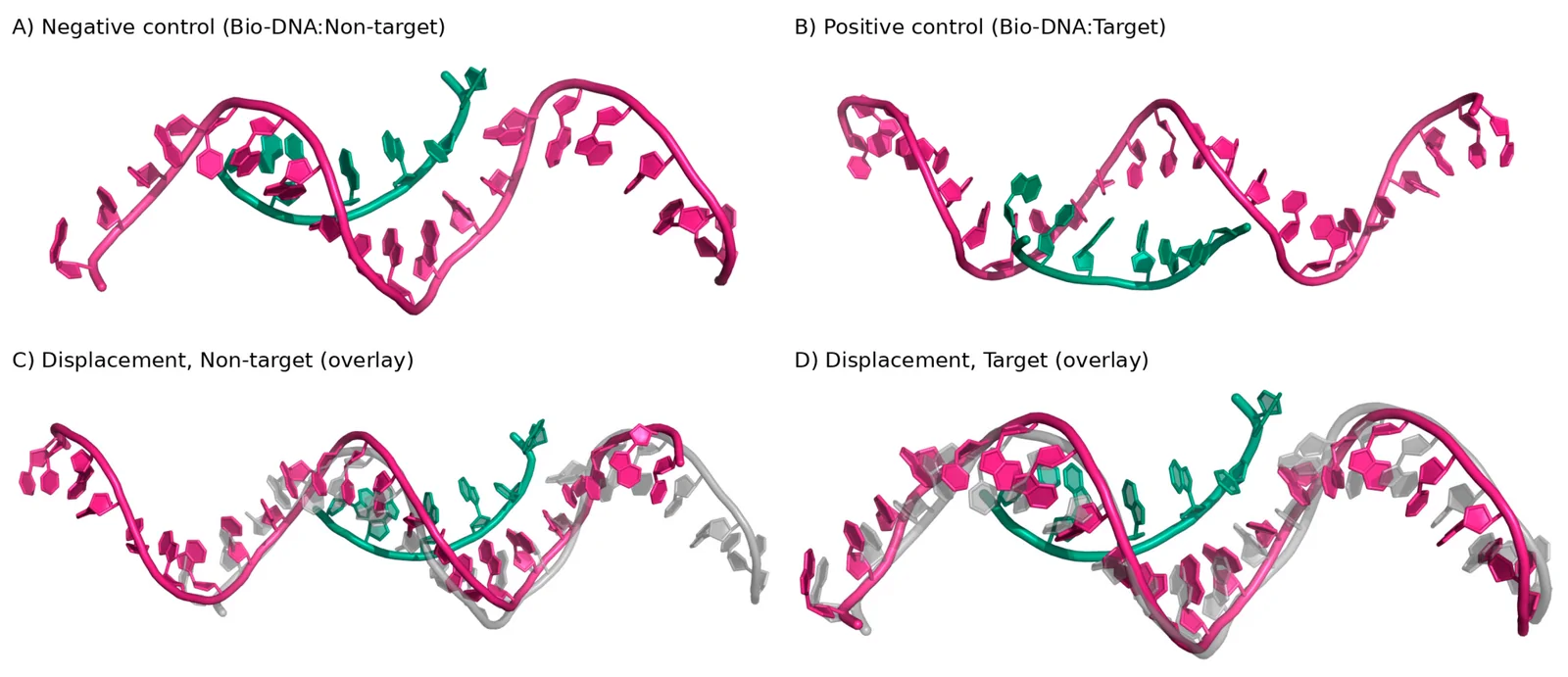
PyMOL rendered lowest energy DNA-parameterised HADDOCK-docked structures for the magnetic bead platform, Method 2. (**A**) negative control (Bio DNA with non-target). (**B**) positive control (Bio DNA with target). (**C**) displacement (non-target). (**D**) displacement (target). The capture strand, Bio DNA, is shown in teal. The target or non-target strand is shown in magenta throughout. As in Figure 4, panel (**B**) does not represent a genuinely complementary duplex, since the recognition region of Bio DNA is not complementary to the target sequence, described in Section 2.1. This pose reflects blind docking rather than confirmed sequence-specific binding. In panels (**C**,**D**), the displaced reporter strand is also shown, in transparent grey, to make the displacement event visible. HADDOCK does not dock three strands at once, so this reporter position was not solved jointly with the incoming target or non-target strand. It is a structural overlay, the reporter bound pose from panel (**B**), superimposed onto the capture strand of the displacement pose using the shared capture strand as the alignment reference (RMSD below 0.5 Å). Any apparent overlap between the grey reporter and the magenta incoming strand reflects them competing for the same binding site on the capture strand.

**Figure 7:**
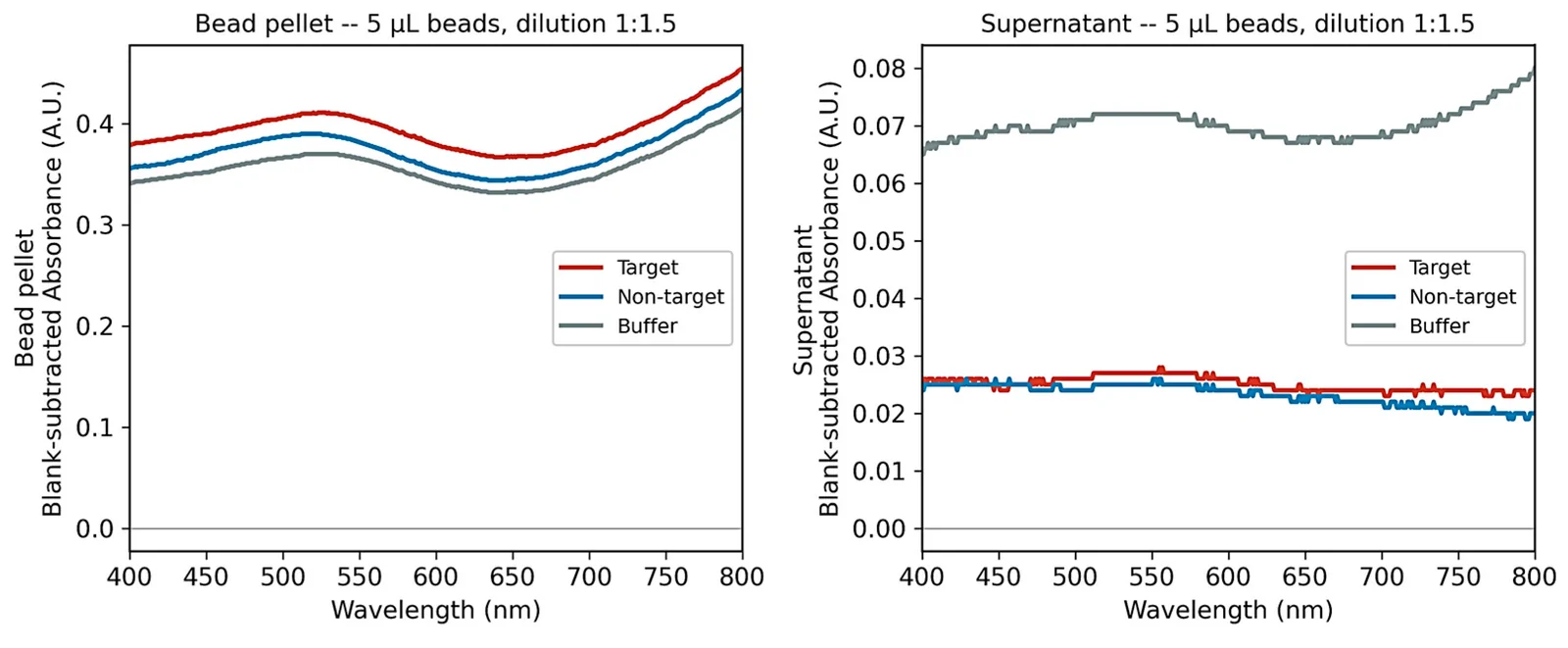
Blank-subtracted ultraviolet-visible (UV-Vis) absorbance spectra, 400 to 800 nm, of the bead pellet (**left**) and supernatant (**right**) fractions, for target (red), non-target (blue), and buffer only (grey) conditions, using the 5 µL bead volume and the least diluted sample tested (1:1.5). The buffer blank, well A1, has been subtracted from every spectrum shown. Three sample dilutions were evaluated (1:10, 1:5, and 1:1.5) across two bead volumes (2 µL and 5 µL). The 1:1.5 dilution with 5 µL beads is shown here as the representative condition (see Section 4.2.7).

**Table 1:** Method 1, electrode platform, High Ambiguity Driven biomolecular DOCKing (HADDOCK) results (RNA-parameterised).

Metric	Negative Control, Capture with Non-Target	Positive Control, Capture with Target	Displacement, Non-Target	Displacement, Target
HADDOCK score	−17.4 ± 4.3	−23.9 ± 2.7	−21.9 ± 2.9	−27.7 ± 5.2
RMSD from lowest energy structure (Å)	18.3 ± 0.7	18.7 ± 0.3	16.0 ± 0.3	1.0 ± 0.6
Z-score	−1.0	−2.3	−1.8	−2.8

Only the HADDOCK score, the root mean square deviation (RMSD) from the lowest energy structure, and the Z-score are shown here. The full energy breakdown, van der Waals energy, electrostatic energy, desolvation energy, restraints violation energy, and buried surface area, are provided in Supplementary Table S1.

**Table 2:** Method 2, magnetic bead platform, HADDOCK results (RNA parameterised).

Metric	Negative Control, Bio DNA with Non-Target	Positive Control, Bio DNA with Target	Displacement, Non-Target	Displacement, Target
HADDOCK score	−27.0 ± 1.1	−26.5 ± 3.5	−27.9 ± 1.9	−38.1 ± 3.2
RMSD from lowest energy structure (Å)	8.2 ± 0.2	6.9 ± 4.0	6.5 ± 0.2	1.3 ± 1.1
Z-score	−1.4	−2.4	−1.2	−1.8

Only the HADDOCK score, the RMSD from the lowest energy structure, and the Z-score are shown here. The full energy breakdown, van der Waals energy, electrostatic energy, desolvation energy, restraints violation energy, and buried surface area are provided in Supplementary Table S2.

**Table 3:** Method 1, electrode platform, HADDOCK results (DNA parameterised).

Metric	Negative Control, Capture with Non-Target	Positive Control, Capture with Target	Displacement, Non-Target	Displacement, Target
HADDOCK score	−63.3 ± 6.8	−62.2 ± 4.2	−61.5 ± 0.8	−72.1 ± 3.1
RMSD from lowest energy structure (Å)	2.8 ± 1.7	25.1 ± 0.2	4.6 ± 1.0	4.1 ± 0.9
Z-score	−1.8	−2.0	−1.6	−2.2

Only the HADDOCK score, the RMSD from the lowest energy structure, and the Z-score are shown here. The full energy breakdown, van der Waals energy, electrostatic energy, desolvation energy, restraints violation energy, and buried surface area are provided in Supplementary Table S3.

**Table 4:** Method 2, magnetic bead platform, HADDOCK results (DNA parameterised).

Metric	Negative Control, Bio DNA with Non-Target	Positive Control, Bio DNA with Target	Displacement, Non-Target	Displacement, Target
HADDOCK score	−41.1 ± 5.5	−25.5 ± 3.8	−31.2 ± 4.8	−55.7 ± 3.8
RMSD from lowest energy structure (Å)	2.3 ± 1.8	1.8 ± 1.0	1.1 ± 0.7	1.1 ± 0.7
Z-score	−1.7	−2.5	−1.5	−2.0

Only the HADDOCK score, the RMSD from the lowest energy structure, and the Z-score are shown here. The full energy breakdown, van der Waals energy, electrostatic energy, desolvation energy, restraints violation energy, and buried surface area are provided in Supplementary Table S4.

## Data Availability

All data are reported in the manuscript and Supplementary Information.

## Supplementary Materials

The following supporting information can be downloaded at: https://www.mdpi.com/article/10.3390/ijms27198652/s1.

## Abbreviations

The following abbreviations are used in this manuscript:

AFM	Atomic force microscopy
AuNP(s)	Gold nanoparticle(s)
B&W buffer	Binding and washing buffer
BIOTEC	National Center for Genetic Engineering and Biotechnology
BSA	Bovine serum albumin
DCN	DCN Diagnostics
DLS	Dynamic light scattering
DNA	Deoxyribonucleic acid
DPV	Differential pulse voltammetry
DSTI	Department of Science, Technology and Innovation
EDTA	Ethylenediaminetetraacetic acid
GPES	General Purpose Electrochemical System
HADDOCK	High Ambiguity Driven biomolecular DOCKing
HEV	Hepatitis E virus
IDT	Integrated DNA Technologies
KMUTT	King Mongkut’s University of Technology Thonburi
KRAS	Kirsten rat sarcoma viral proto-oncogene
MXene	Two-dimensional transition metal carbide/nitride material
NSTDA	National Science and Technology Development Agency
OD	Optical density
PBS	Phosphate-buffered saline
PCR	Polymerase chain reaction
POC	Point-of-care
QCM	Quartz crystal microbalance
RMSD	Root mean square deviation
RNA	Ribonucleic acid
SANBI	South African National Bioinformatics Institute
SEM	Scanning electron microscopy
SPCE	Screen-printed carbon electrode(s)
TCEP	Tris(2-carboxyethyl)phosphine
TEM	Transmission electron microscopy
TIA	Technology Innovation Agency
T_m_	Melting temperature
UV-Vis	Ultraviolet-visible spectrophotometry
UWC	University of the Western Cape
