# Supplementary material for: In Silico Pre-Screening as a Design-Assessment Tool for Gold Nanoparticle-Amplified DNA Displacement Assays: Identifying Sequence-Level Design Limitations Across Electrode and Magnetic-Bead Configurations
Source: Int J Mol Sci. 2026 Sep 27;27(19):8652. doi: 10.3390/ijms27198652 (PMC13665131; doi:10.3390/ijms27198652)
Supplement: Supplementary file 1 [file ijms-27-08652-s001.zip › ijms-4553995-supplementary.pdf]

Supplementary Information

In Silico Pre-Screening as a Design-Assessment Tool for Gold Nanoparticle-Amplified DNA Displacement Assays: Identifying Sequence-Level Design Limitations Across Electrode and Magnetic-Bead Configurations

Darius Riziki Martin [1,2,*], Tatchanun Ngamdee [3], Ruben Cloete [2], Abram Madimabe Madiehe [1,4], Mervin Meyer [1,*] and Patsamon Rijiravanich [3,5]

1 Department of Science, Technology and Innovation (DSTI)/TIA Innovation Platform, Department of
Biotechnology, University of the Western Cape, Bellville 7535, South Africa; amadiehe@uwc.ac.za
2 South African National Bioinformatics Institute (SANBI), University of the Western Cape, Bellville 7535, South Africa; ruben@sanbi.ac.za
3 Sensor Technology Laboratory, Pilot Plant Development and Training Institute, King Mongkut's University of Technology Thonburi, Bang Khun Thian, Bangkok 10150, Thailand; tatchanun.ngam@gmail.com (T.N.); patsamon.rij@biotec.or.th (P.R.)
4 Nanobiotechnology Research Group, Department of Biotechnology, University of the Western Cape,
Bellville 7535, South Africa
5 Industrial Sensor Technology Research Team, National Center for Genetic Engineering and Biotechnology (BIOTEC), National Science and Technology Development Agency (NSTDA), King Mongkut's University of Technology Thonburi, Bang Khun Thian, Bangkok 10150, Thailand
* Correspondence: 3517594@myuwc.ac.za (D.R.M.); memeyer@uwc.ac.za (M.M.)

Supplementary Table S1, Full HADDOCK Energy Breakdown, Method 1, Electrode Platform, RNA Parameterised

Complete HADDOCK energy breakdown for the Method 1 docked complexes reported in main text Table 1, HADDOCK score, RMSD, and Z-score only. Values are mean ± 1 SD, across the top ranked cluster for each condition. These results are from the RNA parameterised docking run described in main text Section 2.1.4. The corresponding DNA parameterised energy breakdown is provided in Supplementary Table S3.

Metric	Negative control	Positive control	Displacement, Non target	Displacement, Target
Van der Waals energy, kcal/mol	-36.1 ± 10.2	-48.4 ± 5.1	-55.8 ± 4.7	-56.9 ± 3.5
Electrostatic energy, kcal/mol	-53.0 ± 48.0	-22.7 ± 33.7	-11.5 ± 10.5	-46.9 ± 43.1
Desolvation energy, kcal/mol	19.4 ± 1.6	20.2 ± 0.6	20.9 ± 1.9	25.9 ± 0.8
Restraints violation energy	98.6 ± 33.1	88.4 ± 66.1	153.2 ± 24.7	126.8 ± 24.4
Buried surface area (Å^2)	1195.7 ± 127.2	1350.6 ± 88.1	1416.5 ± 61.1	1585.4 ± 53.1

Supplementary Table S2, Full HADDOCK Energy Breakdown, Method 2, Magnetic Bead Platform, RNA Parameterised

Complete HADDOCK energy breakdown for the Method 2 docked complexes reported in main text Table 2, HADDOCK score, RMSD, and Z-score only. Values are mean ± 1 SD across the top ranked cluster for each condition. As with Supplementary Table S1, these results are from the RNA parameterised docking run. The corresponding DNA parameterised energy breakdown is provided in Supplementary Table S4. The positive control, Bio DNA with target, condition does not represent a genuinely complementary duplex, described in main text Section 2.1. See main text Figure 4 caption.

Metric	Negative control	Positive control	Displacement, Non target	Displacement, Target
Van der Waals energy, kcal/mol	-40.4 ± 3.2	-41.3 ± 4.7	-35.8 ± 5.7	-46.2 ± 3.7
Electrostatic energy, kcal/mol	-80.2 ± 13.2	-80.0 ± 17.9	-99.0 ± 14.8	-132.2 ± 24.6
Desolvation energy, kcal/mol	26.4 ± 2.2	26.1 ± 3.0	24.1 ± 3.7	28.9 ± 2.2
Restraints violation energy	29.8 ± 15.7	47.3 ± 38.0	35.8 ± 39.8	57.1 ± 27.8
Buried surface area (Å^2)	1136.4 ± 85.1	1120.5 ± 152.1	1122.5 ± 155.9	1243.5 ± 106.0

Supplementary Table S3, Full HADDOCK Energy Breakdown, Method 1, Electrode Platform, DNA Parameterised

Complete HADDOCK energy breakdown for the Method 1 DNA parameterised docked complexes reported in main text Table 3, HADDOCK score, RMSD, and Z-score only. Values are mean ± 1 SD across the top ranked cluster for each condition. These results are from the DNA parameterised docking run described in main text Section 2.1.4 and Section 3.2.1.

Metric	Negative control	Positive control	Displacement, Non target	Displacement, Target
Van der Waals energy, kcal/mol	-44.0 ± 5.7	-42.9 ± 4.9	-46.3 ± 2.3	-51.3 ± 4.5
Electrostatic energy, kcal/mol	-214.1 ± 13.3	-186.0 ± 14.4	-188.5 ± 6.8	-206.2 ± 30.2
Desolvation energy, kcal/mol	22.2 ± 0.5	17.6 ± 2.5	22.1 ± 1.9	20.2 ± 1.7
Restraints violation energy	14.2 ± 18.8	2.9 ± 1.2	4.3 ± 3.8	2.1 ± 1.6
Buried surface area (Å^2)	1678.8 ± 123.7	1554.3 ± 133.6	1654.7 ± 58.5	1663.1 ± 132.1

Supplementary Table S4, Full HADDOCK Energy Breakdown, Method 2, Magnetic Bead Platform, DNA Parameterised

Complete HADDOCK energy breakdown for the Method 2 DNA parameterised docked complexes reported in main text Table 4, HADDOCK score, RMSD, and Z-score only. Values are mean ± 1 SD across the top ranked cluster for each condition. As with Supplementary Table S3, these results are from the DNA parameterised docking run described in main text Section 2.1.4 and Section 3.2.1. The positive control, Bio DNA with target, condition does not represent a genuinely complementary duplex, described in main text Section 2.1. See main text Figure 6 caption.

Metric	Negative control	Positive control	Displacement, Non target	Displacement, Target
Van der Waals energy, kcal/mol	-37.4 ± 3.5	-29.7 ± 2.6	-29.6 ± 3.2	-43.5 ± 6.0
Electrostatic energy, kcal/mol	-117.6 ± 42.8	-74.0 ± 12.2	-93.0 ± 8.4	-156.9 ± 16.8
Desolvation energy, kcal/mol	11.1 ± 1.4	11.4 ± 1.5	8.6 ± 1.6	11.2 ± 2.1
Restraints violation energy	86.8 ± 31.8	76.0 ± 15.8	84.6 ± 29.8	80.9 ± 21.4
Buried surface area (Å^2)	1000.1 ± 50.6	806.9 ± 35.7	963.4 ± 56.1	1054.6 ± 65.9

Supplementary Table S5, Bare Electrode DPV Summary, Magnetic Bead Platform Pellets, 5 µL Condition

Summary values from the 5 µL differential pulse voltammetry traces, referred to here as DPV, for the magnetic bead platform, Method 2, pellet fraction, target, non-target, and buffer only pellet conditions, tested directly on bare electrodes. See Supplementary Note S2, Supplementary Note S3, and Supplementary Figure S4 for full detail.

Condition (5 µL)	Endpoint current (A)	Start current (A)	Δ current (A)
Target	$2.39e^{-6}$	$4.15e^{-7}$	$1.975e^{-6}$
Target, repeat	$1.90e^{-6}$	$4.43e^{-7}$	$1.457e^{-6}$
Non target	$1.93e^{-6}$	$3.06e^{-7}$	$1.624e^{-6}$
Buffer	$2.39e^{-6}$	$6.40e^{-7}$	$1.750e^{-6}$

Supplementary Figure S1, Schematic Model of AuNP-DNA2 Conjugate Architecture

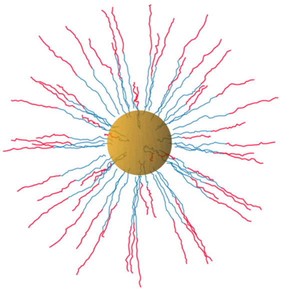

Supplementary Figure S1. *Schematic geometric model of a 40 nm AuNP core carrying approximately 60 thiolated DNA2 strands (blue, T20 spacer; red, recognition segment), showing expected surface density based on literature loading data for non-PEG, non-sonicated, salt-aged conjugates. Illustrative only. Not a physics-based structural prediction.*

This section presents the full results of the electrode-based displacement assay, main text Sections 2.6 to 2.8, for both capture probe immobilisation strategies. These were Method A, electric current assisted deposition, and Method B, passive overnight incubation under humid conditions. Method A had four to five replicates and Method B had two, both below the five or more standard used in the cited literature, and these are the final replicate numbers for this study. The main manuscript, Section 4, discusses possible contributing factors to this variability.

Supplementary Note S1, Electrode-Based Displacement Assay, Methods A and B, Full Results

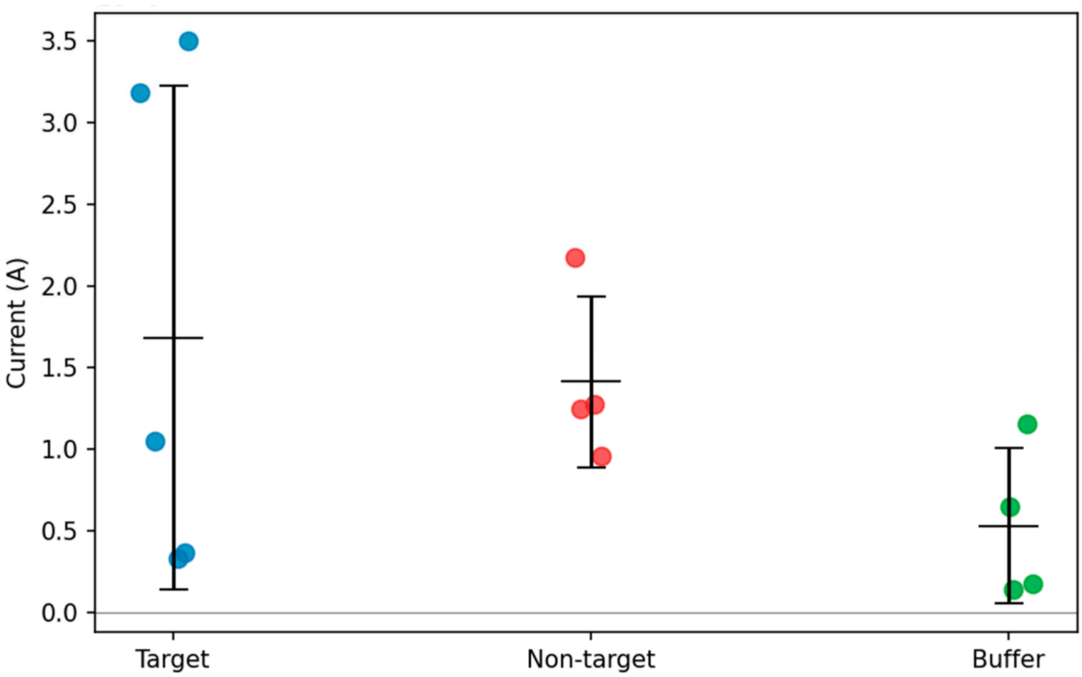

Supplementary Figure S2. *Method A, current assisted, individual electrode replicates for target, non-target, and buffer conditions, with mean ± 1 SD overlaid, n = 4 to 5.*

Mean current was highest for target, 1.69×10^{-6} A, followed by non-target, 1.41×10^{-6} A, and buffer, 5.30×10^{-7} A. Given the small number of replicates and the substantial variance within the target condition, a Welch t test found no statistically significant difference between target and non-target, $p = 0.73$, or between target and buffer, $p = 0.18$. A borderline difference was found between non-target and buffer, $p = 0.05$, with non-target current elevated relative to buffer. This is not the pattern expected for a clean specificity control, and it is discussed further in the main manuscript, Section 4.

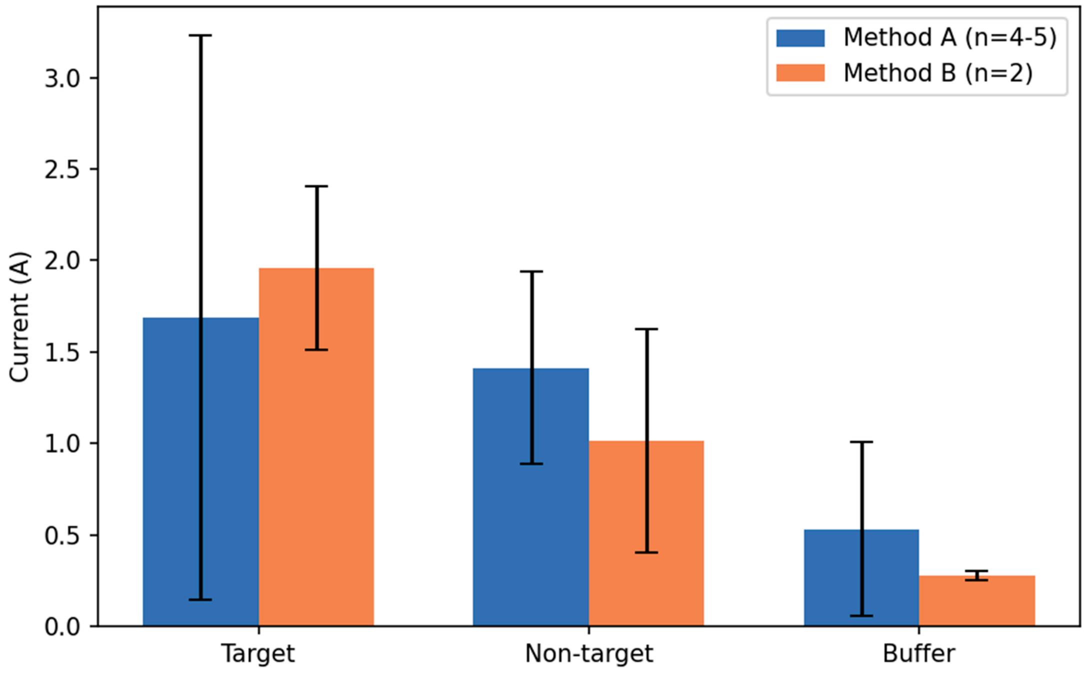

Supplementary Figure S3. *Side by side comparison of Method A, n = 4 to 5, and Method B, n = 2, electrochemical current response for target, non-target, and buffer conditions, reported in mean ± SD (for Method A: n = 4-5). While for Method B, was considered descriptive only, no SD was reported for this sample size (only individual values shown, since n = 2).*

For Method B, we spotted 10 µL of 10 µM capture probe, DNA1, onto the working electrode and incubated it overnight at 4 °C in a humidified container. As with Method A, electrodes were then blocked with 0.1% BSA and washed with 0.1% Tween 20 in 1× PBS, before the hybridisation and displacement steps.

Mean current for Method B was again highest for target, 1.96×10^{-6} A, followed by non target, 1.01×10^{-6} A, and buffer, 2.75×10^{-7} A, n = 2 for all three conditions. Given the very limited statistical power at this sample size, we report these comparisons descriptively rather than with formal p values. Target current was numerically higher than non-target current, which was in turn numerically higher than buffer current, matching the same directional trend seen for Method A, but with only two replicates per condition, this pattern should be treated as descriptive only and not as a confirmed statistical result or a confirmed advantage for either method.

Both methods currently have replicate numbers below the five or more standard used in the cited literature. Method A had four to five replicates and Method B had two. We note this as a limitation in the main manuscript, Section 4.

Supplementary Note S2, Bare Electrode Negative Control, Magnetic Bead Platform Pellets

As a negative control for the magnetic bead platform, Method 2, we tested the bead platform pellet fraction remaining after target-induced displacement and magnetic separation, described in main text Sections 2.9 and 3.3.2, directly on bare, unmodified screen-printed carbon electrodes. We used two pellet volumes, 2 and 5 µL, made up to 100 µL with measuring buffer, matching the main assay. These tests assess whether the pellet retains any AuNP conjugate-associated redox signal after displacement and separation, for target DNA, DNA3, non-target DNA, and measuring buffer-only pellet conditions. We performed a repeat

measurement for the target condition at 5 μL to check run to run reproducibility. Full potential current traces are shown in Supplementary Figure S4. Raw data are available from the corresponding author on reasonable request.

Across all conditions and both pellet volumes, current rose approximately without a discrete Faradaic peak, and target, non-target, and buffer only pellet traces were not distinguishable from one another (see Supplementary Note S3 for the 5 μL summary comparison). This shows that the pellet fraction retained no detectable AuNP conjugate redox signal after displacement and magnetic separation. This is consistent with the AuNP conjugate having already migrated into the supernatant fraction rather than remaining pellet bound. The UV-Vis absorbance data, main text Section 2.2.2, Figure 7, are compatible with that interpretation, but they do not verify it, and they do not demonstrate target-specific displacement. The buffer only condition gave the highest supernatant absorbance of the conditions tested, which is compatible with redistribution or non-specific release of the AuNP conjugate. We report this result here as supplementary, exploratory data rather than in the main manuscript.

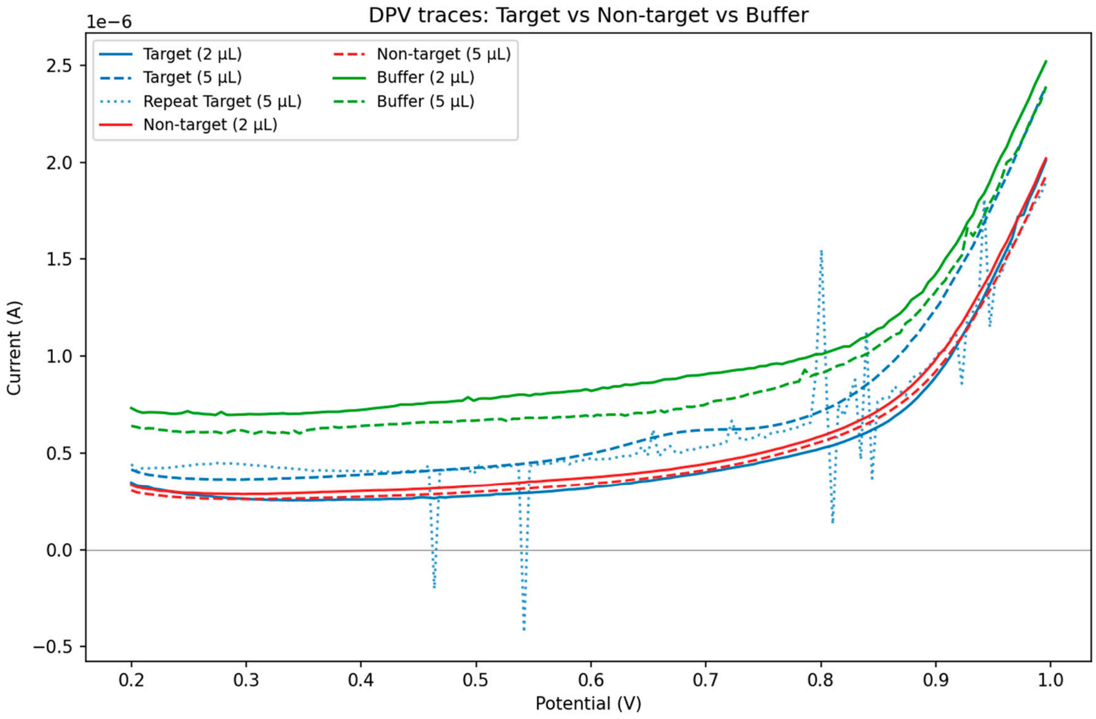

Supplementary Figure S4. *Differential pulse voltammograms for the magnetic bead platform (Method 2). The pellet fraction, tested directly on bare electrodes at 2 and 5 μL pellet volume, following target induced displacement and magnetic separation, for target, DNA3, non-target DNA, and measuring buffer only pellet conditions. An additional repeat run for target at 5 μL is included to show run to run variability. Traces rise approximately without a discrete peak, and target, non-target, and buffer conditions are not distinguishable, showing no detectable AuNP conjugate redox signal remained in the pellet.*

Supplementary Note S3, Magnetic Bead Pellet Bare-Electrode Control: 5 μL Summary Metrics

Because the traces lack a discrete peak, we examined two candidate summary metrics for the 5 μL condition, the volume used in the main assay. These were the endpoint, maximum, current, and the current

rise, Δ, from the start to the end of the scanned potential window. Values are summarised in Supplementary Table S5 and Supplementary Figure S5.

Endpoint current did not compare target from buffer. Both reached 2.39×10^{-6} A, with non-target and the target repeat both lower, about 1.9×10^{-6} A. The Δ current metric ranked the target highest, 1.975×10^{-6} A, but the target repeat run was the lowest of all four traces, 1.457×10^{-6} A. The two target replicates did not agree with each other in relative ranking against -target and buffer. Non target and buffer each have only a single measurement at 5 μL, no repeat run was performed for these conditions, so we could not compute a variance or statistical test for them.

These results do not show a discrete, distinguishable AuNP conjugate redox signal in the pellet fraction at either volume tested. This is compatible with the interpretation given in the main manuscript, Sections 2.2.2 and 2.2.3, that the AuNP conjugate had already migrated to the supernatant following the displacement procedure, and it supports the decision to pursue UV-Vis rather than electrochemical detection for monitoring the distribution of the AuNP conjugate on the magnetic bead platform. The UV-Vis data are not evidence of target-specific displacement.

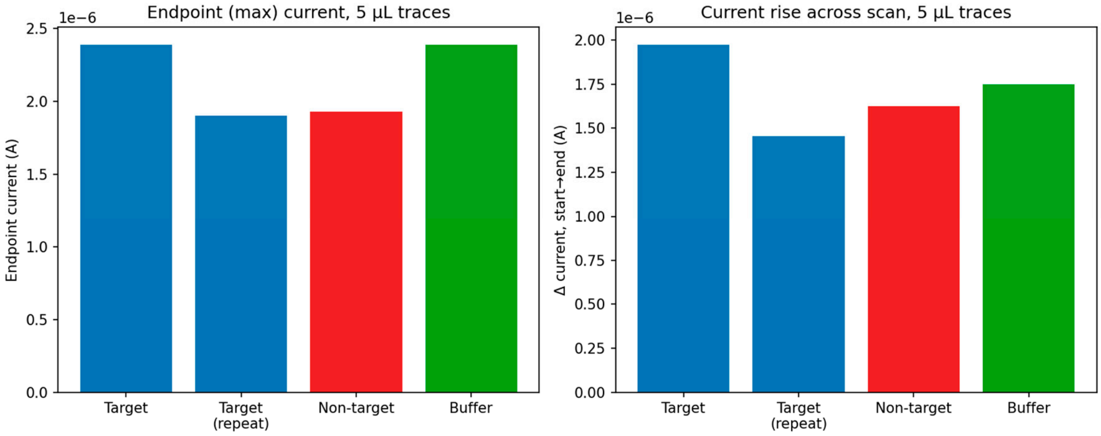

Supplementary Figure S5. *Endpoint, maximum, current (left) and current rise across the scanned potential window (right), for target, target repeat, non-target, and buffer, all at 5 μL sample volume.*

Supplementary Note S4, Derivation of the Approximate Duplex Dissociation Lifetime (~350 Years)

This note provides the full derivation of the duplex dissociation lifetime estimate (~350 years) discussed in main text Section 3.1 (Discussion). All thermodynamic assumptions, parameter sources, and explicit caveats are stated. The estimate is intentionally illustrative. It conveys the thermodynamic stability of the intended duplex and motivates the discussion of strand exchange mechanisms, not to make quantitative predictions about displacement kinetics in the experimental system.

S4.1 Input Parameters

The following parameters were used in the derivation:

Parameter	Value	Source / Justification
**ΔG° (37 °C, 1 M NaCl)**	−22.8 kcal/mol	Nearest-neighbour calculation (SantaLucia 1998 [15]); DNA1

		capture + DNA2/DNA3 recognition region
**Temperature (T)**	310 K (37 °C)	Assay temperature used in experiments
**Gas constant (R)**	1.987×10^{-3} kcal mol^{-1} K^{-1}	Universal gas constant
**k_{on} (DNA hybridisation)**	$\approx 10^{6}$ M^{-1} s^{-1}	Canonical free-solution value for 18-20 nt oligonucleotides (Wetmur & Davidson 1968 [20])
**Standard state (c°)**	1 M	Thermodynamic standard state used in ΔG° calculation

S4.2 Step-by-Step Derivation

Step 1: Equilibrium association constant (K_{eq}):
$\Delta G° = -RT \ln(K_{eq})$
$K_{eq} = \exp(-\Delta G°/RT) = \exp(-(-22.8) / 0.616) = \exp(37.0) \approx 1.17 \times 10^{16}$
(dimensionless at standard state c° = 1 M)

Step 2: Dissociation rate constant (k_{off}):
$K_{eq} = k_{on} / k_{off} \rightarrow k_{off} = k_{on} / K_{eq}$
$k_{off} = 10^{6} / (1.17 \times 10^{16}) \approx 8.5 \times 10^{-11}$ s^{-1}

Step 3: Mean dissociation lifetime (τ):
$\tau = 1 / k_{off} = 1 / (8.5 \times 10^{-11}) \approx 1.18 \times 10^{10}$ s
Converting: 1.18×10^{10} s ÷ (3.156×10^{7} s/year) ≈ 374 years
Reported in main text as ~350 years (rounded to one significant figure)

S4.3 Explicit Caveats and Limitations

The following assumptions are embedded in this estimate. Each introduces uncertainty that limits its applicability to the experimental system:
(1) Free-solution conditions only. The ΔG° and k_{on} values are determined for oligonucleotides in free solution (1 M NaCl). Surface-immobilised DNA1 on the SPCE and AuNP-conjugated DNA2 operate in a substantially different environment. Surface confinement, probe density, and the local ionic environment at the electrode interface are known to reduce k_{on} by 2-100× relative to free solution (Peterson et al. 2001 [21]; Halperin et al. 2004 [22]). This estimate therefore represents an upper bound on surface-duplex stability under assay conditions.
(2) Standard-state concentration assumption. ΔG° is calculated at c° = 1 M. At the experimental DNA concentrations (~10 μM) and assay buffer conditions, the effective equilibrium constants will differ. The estimate is a qualitative illustration of thermodynamic stability, not a quantitative prediction under assay conditions.
(3) Canonical k_{on} assumption. The value $k_{on} \approx 10^{6}$ M^{-1} s^{-1} is a widely cited approximation for unstructured oligonucleotides in free solution (Wetmur & Davidson 1968 [20]). AuNP conjugation and electrode-surface immobilisation may alter this value significantly.
(4) AuNP conjugation effects not included. AuNP conjugation of DNA2 creates a multivalent DNA display on the nanoparticle surface, which can cooperatively stabilise or destabilise hybridisation with DNA1 relative to the free-strand case. These cooperative effects are not captured in the present estimate.
(5) Mechanistic interpretation. The ~350-year estimate motivates the discussion of terminal fraying as a plausible displacement pathway. This mechanism should be treated as a thermodynamically motivated hypothesis rather than an established conclusion. Direct kinetic measurement of strand displacement under assay conditions would be required to validate this interpretation.

Supplementary Table S6, Cohen's d Effect Sizes for Method A Pairwise Electrode Comparisons (Welch t-tests, Bonferroni-corrected α = 0.017)

Effect sizes (Cohen's d) were not computed for this dataset. With n = 4-5 independent electrode replicates per condition for Method A, pooled standard deviation estimates are insufficiently stable to report Cohen's d reliably. The directional

trend (target > non-target > buffer) is reported in main text Section 2.2.1, and the Bonferroni-corrected p-values confirm that no comparison reached statistical significance ($\alpha_{corrected}$ = 0.017). The individual replicate-level current measurements are shown in Supplementary Figure S2.

Supplementary Table S7, Bead Pellet UV-Vis Absorbance at 520 nm (Blank-Subtracted)

Bead pellet fraction, blank-subtracted absorbance at 520 nm, for 2 µL and 5 µL bead volumes across three sample dilutions (1:10, 1:5, 1:1.5). Values are single-well measurements; four tube replicates were prepared per condition and pooled for UV-Vis analysis as described in main text Section 4.2.7. Note: target, non-target, and buffer are of comparable magnitude in the pellet fraction, consistent with non-specific AuNP adsorption rather than sequence-specific displacement.

Bead volume	Dilution	Target (A.U.)	Non-target (A.U.)	Buffer (A.U.)
2 µL	1:10	0.063	0.058	0.054
2 µL	1:5	0.152	0.143	0.136
2 µL	1:1.5	0.318	0.301	0.285
5 µL	1:10	0.068	0.063	0.058
5 µL	1:5	0.161	0.152	0.143
5 µL	1:1.5	0.411	0.390	0.370

Supplementary Table S8, Supernatant UV-Vis Absorbance at 520 nm (Blank-Subtracted)

Supernatant fraction, blank-subtracted absorbance at 520 nm, for 2 µL and 5 µL bead volumes across three sample dilutions (1:10, 1:5, 1:1.5). Values are single-well measurements; four tube replicates were prepared per condition and pooled for UV-Vis analysis as described in the main text Section 4.2.7. Note: buffer alone gave the highest supernatant absorbance, the opposite of the expected pattern for successful target-induced displacement, consistent with the sequence mismatch identified in Section 2.1.

Bead volume	Dilution	Target (A.U.)	Non-target (A.U.)	Buffer (A.U.)
2 µL	1:10	0.008	0.007	0.018
2 µL	1:5	0.014	0.012	0.032
2 µL	1:1.5	0.019	0.017	0.041
5 µL	1:10	0.008	0.007	0.018
5 µL	1:5	0.013	0.011	0.028
5 µL	1:1.5	0.027	0.025	0.072

Supplementary Data File S1, Input Sequences, HADDOCK Run Conditions, and mfold/RNAstructure Calculation Parameters

This file documents all input sequences (in FASTA format), HADDOCK 2.4 web server run conditions, and mfold secondary-structure calculation parameters used in this study, to enable full reproducibility of the computational workflow described in main text Sections 4.1.1-4.1.4. Actual HADDOCK run IDs are to be inserted by the corresponding author from job records on the HADDOCK 2.4 server (https://wenmr.science.uu.nl/haddock2.4/).

S1.1 Oligonucleotide Input Sequences

The following five sequences were used in the thermodynamic, secondary-structure, and docking analyses. Sequences are given 5'→3'. Modification codes: thiol- = 3'-thiol modification; Biotin- = 5'-biotin; T_{20} = poly-thymine spacer of 20 nt.

Label	Role	Sequence (5'→3')	Source / Notes
**DNA1**	Capture probe (electrode platform)	5'-T_{20}-CGTTTACCCAGTCACGAC-3'	Stated in main text Section 4.2.4. T_{20} = 20-thymine spacer; 18

			nt target-complementary recognition region.
**DNA2**	AuNP-conjugated reporter probe	5'-thiol-GTCGTGACTGGGTAAACG-3'	Reverse complement of DNA1 recognition region (18 nt). Sequence-identical to DNA3 (target). Thiol modification enables AuNP conjugation.
**DNA3**	Target sequence (displacement analyte)	5'-GTCGTGACTGGGTAAACG-3'	Sequence-identical to DNA2 (reporter). Confirmed in main text Section 3.1.
**Non-target**	Negative control (electrode & bead platforms)	5'-CCGGCGGTGGTTTCTGGGGTGAC-3'	Stated in main text Section 4.2.6. Linear target DNA from published HEV assay [4]. 23 nt; unrelated to DNA1 recognition region.
**Bio DNA (original: MISMATCH)**	Bead capture probe (mismatched: used in experiment)	5′-Biotin-spacer-T20-GCGGGA-3′ (recognition region; full construct as supplied by IDT)	Recognition region GCGGGA (6 nt); shares at most a 3-nt overlap with the AuNP thiol DNA conjugate. Mismatch identified computationally (Section 2.1.1).
**Bio DNA (corrected: proposed)**	Proposed corrected bead capture probe (untested)	5'-Biotin-spacer-T_{20}-CGTTTACCCAGTCACGAC-3'	Stated in main text Sections 2.1.1 and 6 (Future Work). Shares full 18 nt recognition region with DNA1. Not experimentally validated in this study.

S1.2 HADDOCK 2.4 Web Server: Run Conditions (16 Total Runs)

All docking runs were performed using the HADDOCK 2.4 web server (https://wenmr.science.uu.nl/haddock2.4/), EASY access level, with all settings left on default unless otherwise specified. Eight runs used the server default RNA force-field parameterisation; eight were repeated using explicit DNA residue coding (DA/DC/DG/DT). Active residues: full recognition region of the capture strand (DNA1 or Bio DNA). Interacting partner: entire complementary strand treated as interacting. Clustering: default HADDOCK 2.4 method; maximum 200 models; top 10 clusters reported; top-ranked cluster selected for analysis. Actual run IDs must be entered by the corresponding author from HADDOCK server job records.

Run No.	Platform	Condition	Parameterisation
**1**	Method 1 (Electrode)	Negative control (DNA1 + Non-target)	RNA (default)
**2**	Method 1 (Electrode)	Positive control (DNA1 + DNA2 Reporter)	RNA (default)

**3**	Method 1 (Electrode)	Displacement Non-target (DNA1 + Non-target overlay)	RNA (default)
**4**	Method 1 (Electrode)	Displacement Target (DNA1 + DNA3 Target overlay)	RNA (default)
**5**	Method 2 (Bead)	Negative control (Bio DNA + Non-target)	RNA (default)
**6**	Method 2 (Bead)	Positive control (Bio DNA + DNA2 Reporter)	RNA (default)
**7**	Method 2 (Bead)	Displacement Non-target (Bio DNA + Non-target overlay)	RNA (default)
**8**	Method 2 (Bead)	Displacement Target (Bio DNA + DNA3 Target overlay)	RNA (default)
**9**	Method 1 (Electrode)	Negative control (DNA1 + Non-target)	DNA (DA/DC/DG/DT)
**10**	Method 1 (Electrode)	Positive control (DNA1 + DNA2 Reporter)	DNA (DA/DC/DG/DT)
**11**	Method 1 (Electrode)	Displacement Non-target (DNA1 + Non-target overlay)	DNA (DA/DC/DG/DT)
**12**	Method 1 (Electrode)	Displacement Target (DNA1 + DNA3 Target overlay)	DNA (DA/DC/DG/DT)
**13**	Method 2 (Bead)	Negative control (Bio DNA + Non-target)	DNA (DA/DC/DG/DT)
**14**	Method 2 (Bead)	Positive control (Bio DNA + DNA2 Reporter)	DNA (DA/DC/DG/DT)
**15**	Method 2 (Bead)	Displacement Non-target (Bio DNA + Non-target overlay)	DNA (DA/DC/DG/DT)
**16**	Method 2 (Bead)	Displacement Target (Bio DNA + DNA3 Target overlay)	DNA (DA/DC/DG/DT)

Specific HADDOCK job IDs are available from the corresponding author on reasonable request.

S1.3 mfold Secondary-Structure Calculation Conditions

DNA secondary-structure self-checks were performed using the mfold web server (http://www.unafold.org/mfold/applications/dna-folding-form.php) for all five oligonucleotide sequences independently. The following conditions were applied consistently across all calculations:

Parameter	Setting Used	Justification
**Folding mode**	DNA	All assay sequences are DNA oligonucleotides
**Temperature**	37 °C	Matching the assay experimental temperature
**[Na^+]**	1.0 M	Default mfold ionic condition (standard state)
**[Mg^{2+}]**	0 mM	Default (no magnesium specified)
**Maximum structures**	50	Default; sufficient to assess dominant fold
**Window parameter**	Default (W=0)	Default mfold setting

Results are reported as the optimal (lowest) free energy (ΔG, kcal/mol) for self-folding of each individual strand at 37 °C. The criterion applied: any ΔG value more negative than −5 kcal/mol was considered a potentially competing self-structure requiring further assessment (none of the five sequences met this criterion; see Figure 2 in main text and main text Section 2.1.2).

Note: Nearest-neighbour duplex thermodynamic parameters (ΔG and Tm for each capture-partner pair) were calculated separately using the SantaLucia unified parameter set [15], at 37 °C and 1 M NaCl standard state, as described in main text Section 4.1.1 and Supplementary Note S4.
